# Comparative efficacy of various exercise types and doses for quality of life in patients with heart failure: a network and dose–response meta-analysis

**DOI:** 10.3389/fcvm.2026.1774345

**Published:** 2026-03-25

**Authors:** Haotian Jin, Yinan Zhang, Xinmiao Feng, Yi Sun

**Affiliations:** 1 Department of Humanities and Social Science, Beijing Electronic Science and Technology Institute, Beijing, China; 2 Chinese Wushu College, Beijing Sports University, Beijing, China

**Keywords:** dose–response, exercise, heart failure, network meta-analysis, quality of life

## Abstract

**Background:**

Heart failure (HF) significantly compromises quality of life (QoL). Although exercise interventions are a well-established strategy for improving QoL in patients with HF, the efficacy of various exercise modalities and effective doses remains unclear. This study aims to ascertain the efficacy of different exercise modalities and their relative doses in optimizing QoL in patients with HF.

**Methods:**

This systematic review and network meta-analysis searched PubMed, Web of Science, Embase, MEDLINE, and Cochrane databases. Randomized controlled trials (RCTs) involving exercise interventions were included. Data on dose metrics (METs-min/week), treatment regimens, exercise intensity, and study duration were extracted and analyzed.

**Results:**

Thirty RCTs involving 1,780 participants were included in the review. High-intensity interval training (HIIT), combined aerobic and resistance training (CT), mind–body exercise (MBE), and resistance training (RT) all demonstrated substantial efficacy in improving QoL compared with the control group (SMD = −0.59, −0.57, −0.58, and −0.57; SUCRA: 64.8%, 63.9%, 62.1%, and 62%, respectively). The optimal doses were approximately 750, 1,000, 390, and 490 METs-min/week for HIIT, CT, MBE, and RT, respectively. While aerobic exercise (AE) also demonstrated efficacy, the observed therapeutic benefit was modest.

**Conclusion:**

HIIT, CT, MBE, and RT exhibit strong and comparable efficacy in improving QoL in patients with HF, whereas AE offers a more modest benefit. MBE demonstrates efficacy at relatively low doses, while CT and HIIT yield progressive improvements with increasing doses.

**Systematic Review Registration:**

https://www.crd.york.ac.uk/PROSPERO/view/CRD420251181909, PROSPERO CRD420251181909.

## 1 Introduction

Heart failure (HF) is a major global health challenge that significantly increases the risk of poor quality of life (QoL), physical frailty, prolonged recovery, and recurrent hospital admissions (1, 2). Associated with long-term physical and mental health consequences, HF often leads to substantial morbidity and mortality (3, 4). Despite the availability of various therapeutic interventions, HF continues to impose a heavy burden on physical functioning, psychosocial well-being, and activities of daily living (5, 6). Consequently, improving QoL has become an important therapeutic goal, particularly because many patients prioritize QoL over longevity (7, 8).

Exercise has long been established as a cornerstone of non-pharmacological therapy in HF management (9, 10). Evidence from randomized controlled trials (RCTs) and meta-analyses indicates that exercise interventions can enhance functional capacity, alleviate symptoms, and significantly improve QoL in patients with both reduced and preserved ejection fraction (11–13). However, the comparative efficacy and optimal doses for improving QoL across diverse exercise modalities, including high-intensity interval training (HIIT), combined aerobic and resistance training (CT), resistance training (RT), aerobic exercise (AE), and mind–body exercise (MBE), remain unclear. While some studies suggest a positive dose–response relationship, others caution about diminishing returns and the risk of overexertion (14, 15).

To fill these gaps, this study presents a comprehensive network and dose–response meta-analysis. It compares the relative efficacy of different exercise modalities and identifies the effective exercise doses for improving QoL in patients with HF. The findings provide critical evidence regarding exercise type, intensity, and dosage, supporting precision exercise prescriptions and advancing HF rehabilitation strategies.

## 2 Methods

This meta-analysis was conducted in accordance with the Preferred Reporting Items for Systematic Reviews and Meta-Analyses extension for network meta-analyses (PRISMA-NMA) and the guidelines outlined in the Cochrane Handbook. The review was registered (PROSPERO: CRD420251181909) and reported in accordance with the PRISMA checklist.

### 2.1 Search strategy

A comprehensive search was conducted across PubMed, Web of Science, Embase, MEDLINE, and the Cochrane Library databases from their inception to March 2025. Medical Subject Headings (MeSH) terms related to exercise, heart failure, and quality of life were used. These terms were combined using Boolean operators (“AND” “OR”). In addition, the reference lists of relevant articles were manually screened. Two investigators independently performed title, abstract, and full-text screening. Discrepancies were resolved through discussion with a third reviewer.

### 2.2 Eligibility criteria

The inclusion criteria followed the PICOS standard (16): (1) participants (≥18 years) with HF across the full spectrum of left ventricular ejection fraction (LVEF), including reduced (HFrEF, LVEF ≤40%), mildly reduced (HFmrEF, LVEF 41%–49%), and preserved ejection fraction (HFpEF, LVEF ≥50%); (2) exercise interventions divided into five types; (3) control groups including no intervention, usual care, wait-list, maintenance of daily activities, attention control, guideline-based control, and health education; (4) outcomes measuring QoL; and (5) published RCTs (e.g., individual, cluster, or first-phase crossover designs).

Exclusion criteria were as follows: (1) acute (single-session) interventions; (2) indistinct exercise modalities/doses; (3) absence of baseline QoL assessment; (4) QoL outcomes that could not be assessed using change scores; and (5) studies involving participants with a mean age <18 years.

### 2.3 Data extraction

Data extraction was performed independently by two investigators. They screened the studies that met the inclusion standards and resolved any discrepancies through discussion with a third author. The extracted data included study characteristics (author information, publication year, age, gender, number of participants, physical condition, baseline information, adherence), intervention characteristics (type, frequency, duration, session, time, and intensity), and QoL outcomes. QoL data were extracted from the following validated instruments: the Minnesota Living with Heart Failure Questionnaire (MLHFQ), the Short Form-36 (SF-36), the Kansas City Cardiomyopathy Questionnaire (KCCQ), and the Icelandic Quality of Life Questionnaire (Heilsutengd lífsgæði). We standardized the directionality of the scores to ensure consistency across all analyses. In our final analysis, a lower SMD consistently indicated better QoL.

Intervention intensity and exercise dosage were calculated based on the American College of Sports Medicine (ACSM) guidelines (e.g., low: 9–11 Borg RPE, 57%–64% HR max or 30%–50% 1RM; moderate: 12–13 Borg RPE, 64%–76% HR max or 50%–70% 1RM; vigorous: 14–17 Borg RPE, 76%–96% HR max or 70%–85% 1RM), and the MET values for relative intensity ranges were used to calculate the weekly exercise volume. Session duration was extracted as the actual minutes per session reported in each study (17). Weekly volume was calculated as follows: MET value × minutes per session × weekly frequency. For exercises (e.g., Tai chi, Yoga, Qigong) where numerical intensity parameters are typically not reported, we directly used the standardized MET values assigned to these activities in the Compendium of Physical Activities (18).

When standard deviations (SDs) were not reported, they were derived from SEs, CIs, *t*-values, quartiles, ranges, or *p*-values (19). The corresponding author was responsible for handling missing data. Studies were excluded if the data remained unavailable.

### 2.4 Risk of bias

Risk of bias was independently assessed by two reviewers using the Cochrane RoB 2 tool (20). This assessment tool evaluates five domains: randomization process, deviations from the intended interventions, missing outcome data, measurement of the outcome, and selection of the reported result. The overall risk of bias judgment was also determined. Disagreements were resolved through consultation with a third author. The certainty of evidence was assessed using the CINeMA web application (21).

### 2.5 Measures of treatment effect

To assess QoL outcomes, the mean (M), standard deviation (SD), and sample sizes (*n*) were extracted. For studies reporting multiple time points (e.g., midintervention, follow-up, or crossover phases), only the data from the end of the intervention period were analyzed. Exercise interventions were categorized into five modalities: aerobic exercise (AE), high-intensity interval training (HIIT), combined aerobic and resistance training (CT), mind–body exercise (MBE, including Qigong, Yoga, and Tai Chi), and resistance training (RT). Further details on exercise categories are provided in Supplementary 1.5. In addition, interventions were classified based on exercise dosage. Dosage was defined as the total energy expenditure in metabolic equivalents of task (METs), calculated from exercise duration, frequency, and intensity. It was expressed as METs-minutes per week (METs-min/week) (22, 23) and categorized into levels of 0 (control group), 250, 500, 750, or 1,000 METs-min/week to establish network connectivity (24).

### 2.6 Statistical analysis

A frequentist network meta-analysis (NMA) was conducted in Stata (version 17.0) using standardized mean differences (SMDs). Due to differences in measurement scales, data directionality was standardized prior to analysis so that lower values represented better QoL. We used both inverse-variance (i) and common-effect (c) models to calculate general effect estimates. For better visualization, forest plots were generated for both pairwise and network-level treatment effects. Network plots were also constructed to illustrate the geometry and connectivity of comparisons among the various exercise interventions. The ranking probability of interventions was estimated using a random-effects model. In addition, surface under the cumulative ranking (SUCRA) values were calculated to summarize the ranking hierarchy of treatments. Relative effect estimates across all treatment pairs were presented in the Table 1. Tau-squared (*τ*^2^) test and *I*^2^ statistics were used to analyze statistical heterogeneity. In addition, inconsistency was evaluated using the design-by-treatment test (25) and the SIDE (separating indirect from direct evidence) test (26).

**Table 1:** League table of SMD for QoL comparisons across different exercise interventions.

HIIT					
−0.01 (−0.49, 0.46)	CT				
−0.01 (−0.63, 0.61)	0.00 (−0.52, 0.53)	MBE			
−0.01 (−0.66, 0.64)	0.00 (−0.55, 0.55)	−0.00 (−0.69, 0.69)	RT		
−0.10 (−0.52, 0.32)	−0.09 (−0.37, 0.20)	−0.09 (−0.58, 0.40)	−0.09 (−0.60, 0.43)	AE	
**−0.59** **(−1.00, −0.17)**	**−0.57** (**−0.82, −0.32)**	**−0.58** (**−1.04, −0.11)**	**−0.57 (−1.09, −0.06)**	**−0.49 (−0.68, −0.29)**	CON

The analysis indicates that HIIT (SMD = −0.59, 95% CI: −1.00 to −0.17), CT (SMD = −0.57, 95% CI: −0.82 to −0.32), MBE (SMD = −0.58, 95% CI: −1.04 to −0.11), and RT (SMD = −0.57, 95% CI: −1.09 to −0.06) were all effective in improving QoL compared with the control group. In addition, AE demonstrated a moderate effect (SMD = −0.49, 95% CI: −0.68 to −0.29).

To evaluate local inconsistency, the node-splitting method was used. A *p*-value <0.05 indicated significant inconsistency between direct and indirect estimates. In addition, loop-specific inconsistency was assessed using inconsistency factor plots. The 95% confidence interval including zero indicated that the inconsistency was not significant. Publication bias was evaluated using Egger's test and visualized through comparison-adjusted funnel plots (27).

Bayesian dose–response network meta-analysis (MBNMA) was conducted using the MBNMAdose and multinma packages in R (version 4.5, R Foundation for Statistical Computing, Vienna, Austria). MBNMA combines the benefits of conventional network meta-analysis with dose–response models to estimate treatment effects across different doses and agents (28).

A random-effects network meta-analysis model was fitted using the SMD as the effect measure. The connectivity and feasibility of the treatment network were visually assessed at both the treatment and agent levels. Network geometry was evaluated to ensure that all treatments and dose levels were connected (Supplementary 2.1, 2.2). A summary plot showing responses (SMD) across different doses and treatments is presented in Supplementary 2.3.

To identify the optimal model fit, several models were evaluated, including linear, exponential, Emax, restricted cubic splines, and non-parametric monotonically increasing models. A deviance plot was generated for further evaluation (Supplementary 2.4, 2.5) (29, 30). Among these, the restricted cubic splines models demonstrated the best model fit. This approach offers a flexible framework for modeling possible non-linear associations across different doses. Model fit was evaluated using predicted values and graphical summaries overlaid on the observed data.

To evaluate consistency, both the consistency model and the unrelated mean effect (UME) model were assessed by comparing the residual deviance, DIC, and other parameters (Supplementary 2.6). In addition, a plot of posterior deviance distributions was generated for further comparison between the models (Supplementary 2.7).

Local inconsistency was further evaluated using the node-splitting method (31). Density plots (Supplementary 2.8) of the split comparisons were used to assess potential inconsistencies. Further details are provided in Supplementary 2.9.

To explore potential sources of heterogeneity and inconsistency, we conducted network meta-regression analyses using a Bayesian framework implemented in the gemtc and rjags packages in R. We treated mean age and baseline LVEF as continuous covariates to assess their potential impact on the treatment effects (measured as SMD). A shared regression coefficient was assumed across all treatment comparisons. The models were fitted using Markov Chain Monte Carlo (MCMC) simulations with four chains. If the 95% credible interval (CrI) of the regression coefficient did not include zero, the covariate was considered a significant effect modifier.

The dose–response network meta-analysis was conducted using the MBNMAdose package in R. Model fitting was conducted using MCMC methods implemented in JAGS (Just Another Gibbs Sampler), accessed through the rjags interface. Convergence was assessed by visually inspecting trace plots and residual diagnostics. In the absence of prior information, the default prior distributions provided by MBNMAdose were used.

## 3 Results

### 3.1 Study selection and characteristics

A total of 30 studies were included in the meta-analysis, and 27 studies were included in the dose–response analysis (three studies were excluded due to unquantifiable exercise doses). The details of exercise modalities across these studies are as follows: 18 (17) studies on AE, three studies on HIIT, 11 (9) studies on CT, four studies on MBE, and three studies on RT. (The numbers in parentheses represent the number of studies that were included in the dose–response analysis, and the numbers outside the parentheses indicate the number of studies included in the network meta-analysis.)

**Figure 1:**
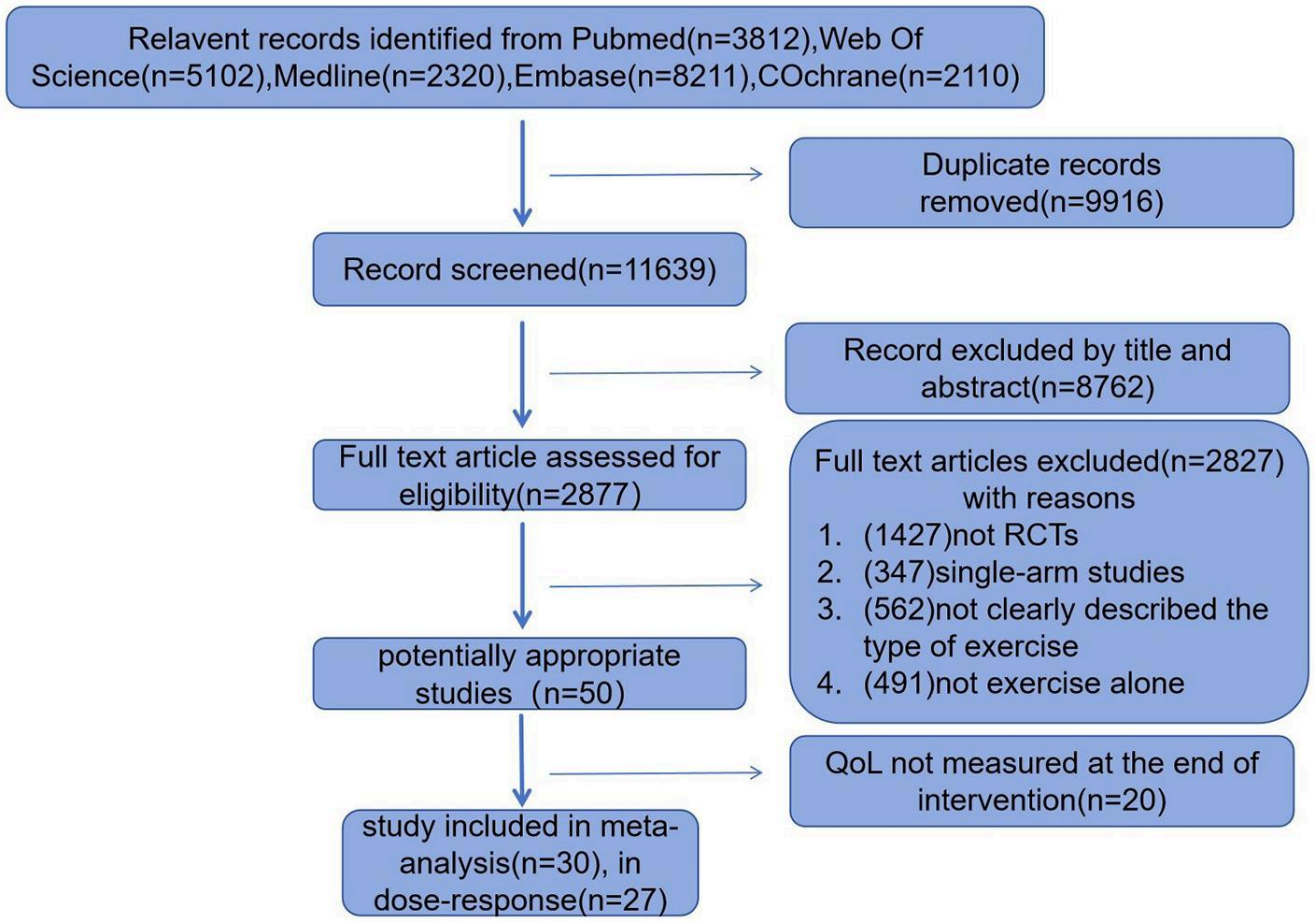
Flowchart of the search process.

Figure 1 illustrates the Preferred Reporting Items for Systematic Reviews and Meta-Analyses (PRISMA) flowchart of the study selection process. After the initial screening of 11,639 studies, 2,877 full-text articles were assessed. Finally, 30 RCTs including five types of exercise interventions were included in the analysis. The search strategy is presented in Supplementary 1.1.

The included studies involved 1,780 participants. The mean age of participants across all included studies was 59.72 years, ranging from 48.00 to 71.44 years. It is worth mentioning that some studies did not adequately report or control for the use of heart failure medications.

The intervention duration ranged from 6 to 36 weeks, and the exercise frequency ranged from 2 to 5 sessions per week. Exercise intensity was tailored to the type of intervention. Additional characteristics of included studies are presented in Supplementary 1.2.

### 3.2 Quality assessment

Thirty RCTs were evaluated for risk of bias. Among these studies, 13 applied the intention-to-treat (ITT) approach, while 17 applied the per-protocol (PP) approach. The majority of ITT studies demonstrated a low risk of bias in the domains of the randomization process (76.9%), deviations from intended interventions (84.6%), and missing outcome data (92.3%). However, the domains of measurement of the outcome (38.5%) and selection of the reported result (76.9%) frequently raised some concerns. Consequently, 69.2% of ITT studies received an overall rating of some concerns.

PP studies demonstrated a low risk of bias in the domains of the randomization process (76.5%) and measurement of the outcome (52.9%). However, the domains of missing outcome data and deviations from intended interventions showed higher proportions of high risk (23.5%–29.4%). Overall, 64.7% of PP studies were rated as some concerns, and 23.5% were rated as high risk. Overall, the majority of studies were rated as some concerns, with only a minority identified as high risk. Further details are provided in Supplementary 1.3.

In addition, we graded the evidence in the network meta-analysis using CINeMA. For the main results, we assessed 86.67% of the evidence as very low in credibility. The CINeMA confidence ratings are presented in Supplementary 1.4.

### 3.3 Network meta-analyses

**Figure 2:**
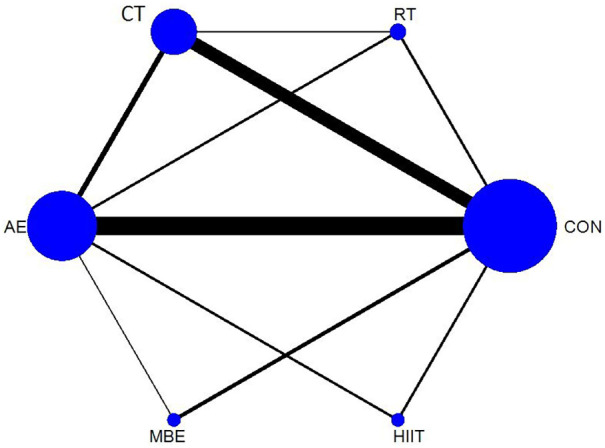
Network plot.

A frequentist network meta-analysis was conducted using a multivariate random-effects consistency model. The network plot is presented in Figure 2. The results showed that all exercise interventions significantly improved QoL compared with the control group (CON). The forest plot is presented in Supplementary 3.1.

Global inconsistency was assessed using the design-by-treatment interaction model. No significant inconsistency was detected between direct and indirect comparisons [*χ*^2^(11) = 13.57, *p* = 0.26]. Subsequently, node-splitting analyses revealed no significant local inconsistency between direct and indirect evidence. Detailed results are provided in Supplementary 3.2.

Egger's test indicated no statistically significant small-study effect (*p* = 0.11), suggesting a low risk of publication bias. The comparison-adjusted funnel plot was generally symmetrical. The funnel plot is presented in Supplementary 3.3. All evaluated loops demonstrated good consistency between direct and indirect estimates (the 95% CI included zero). The loop-specific plot is shown in Supplementary 3.4. Meta-regression did not identify significant effect modifiers, including mean age and LVEF. Detailed meta-regression results are provided in Supplementary 3.5.

**Figure 3:**
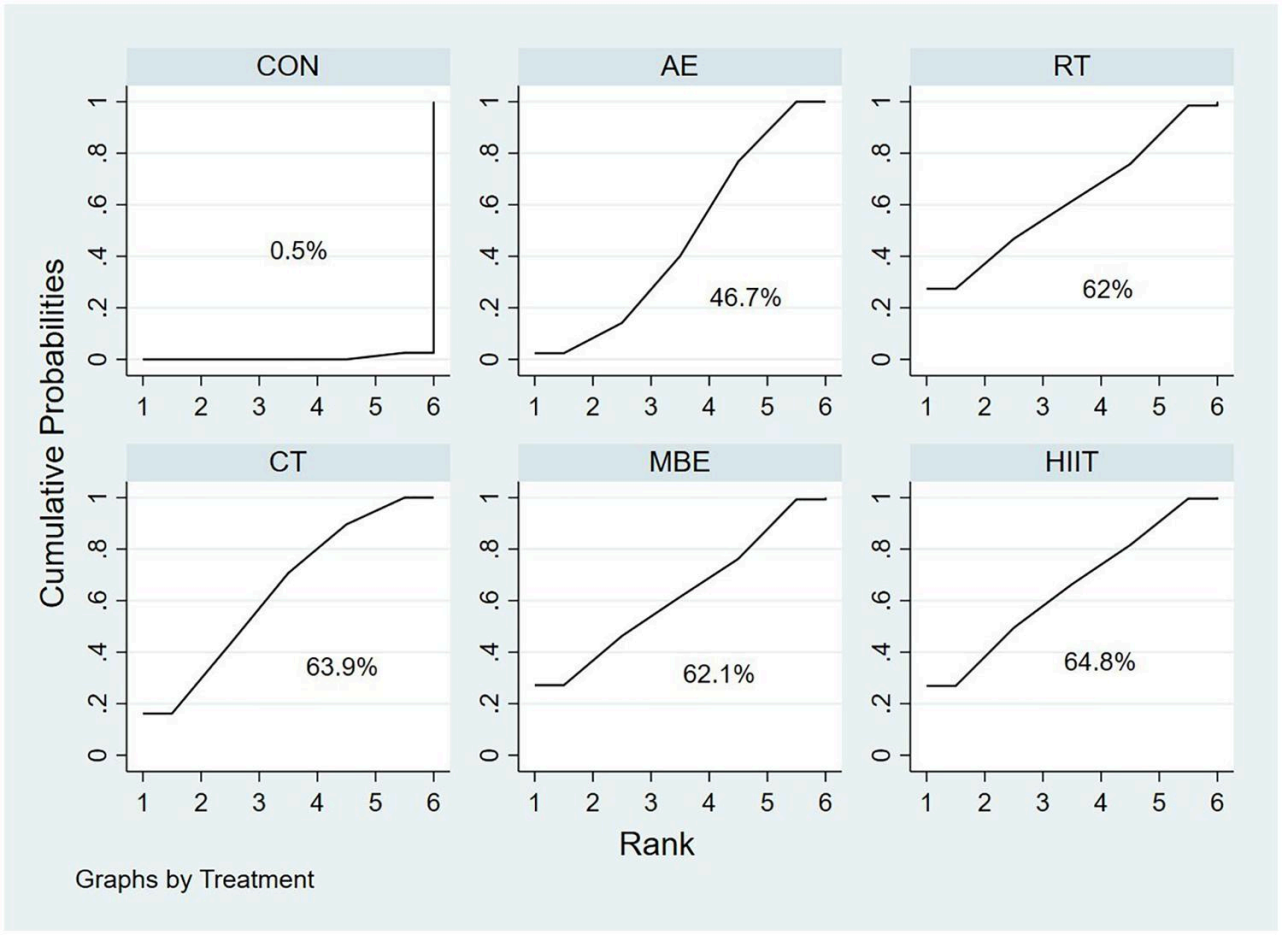
SUCRA for QoL.

Regarding SUCRA values, HIIT, CT, MBE, and RT showed relatively high probabilities of being the most effective interventions for improving QoL (64.8%, 63.9%, 62.1%, and 62.0%, respectively). However, AE exhibited a comparatively lower SUCRA value (46.7%). The SUCRA rankings are shown in Figure 3.

**Figure 4:**
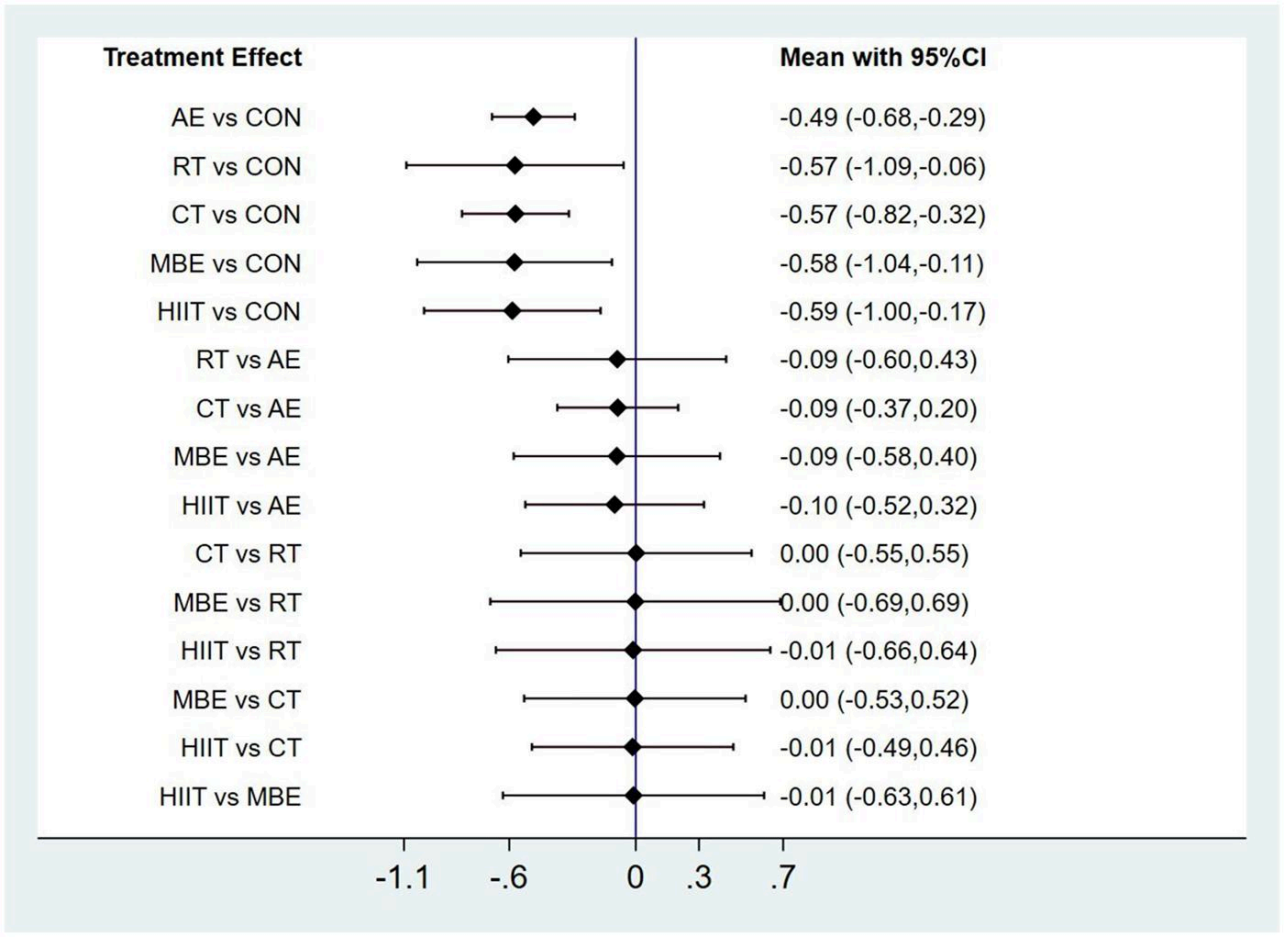
Pairwise comparison forest plot.

In summary, HIIT, CT, MBE, RT, and AE were all effective strategies for improving QoL, although AE demonstrated comparatively lower efficacy. Detailed comparisons between groups are presented in Figure 4.

### 3.4 Dose–response relationships

**Figure 5:**
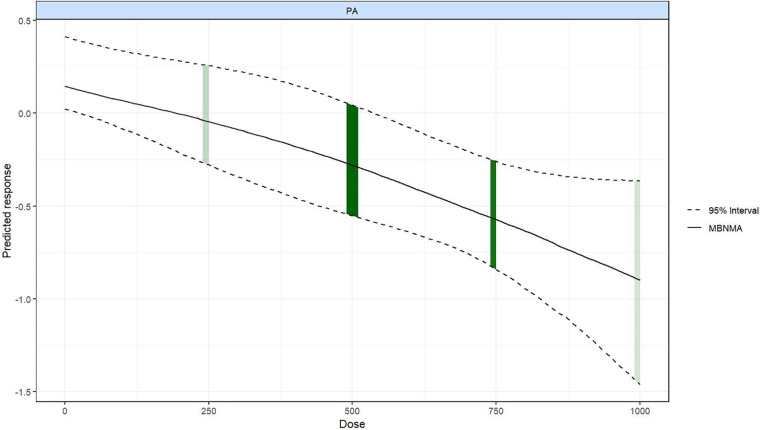
Dose–response relationship between overall exercise dose and QoL.

A steady decline in the predicted response was observed as the exercise dose increased, suggesting that higher doses of PA were related to greater improvements in QoL (Figure 5). The most effective dose was observed at 1,000 METs-min/week (SMD = –0.90; 95% CrI: –1.46 to –0.36). The dose range yielding significant QoL improvement was 540–1,000 METs-min/week.

**Figure 6:**
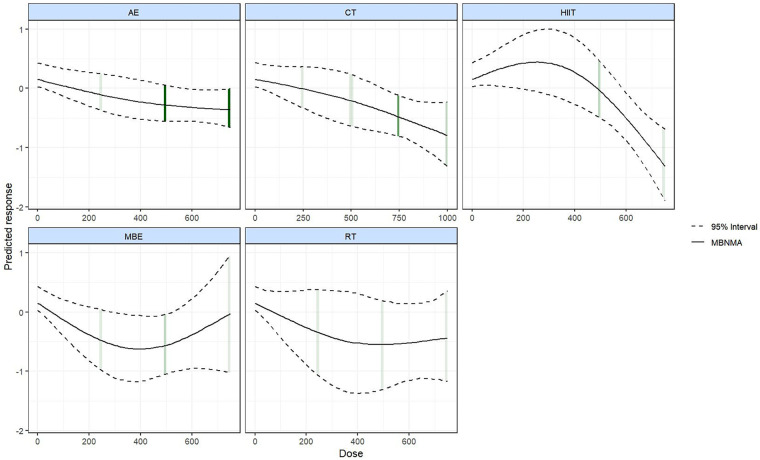
Dose–response relationship among different types of exercises.

Dose–response relationships varied across different exercise modalities. Figure 6 shows that HIIT, CT, MBE, RT, and AE all presented improvements in QoL as the exercise dose increased. However, the association between RT dose and QoL was not statistically significant (95% CrI included the null value). HIIT showed an optimal dose at 750 METs-min/week (SMD = −1.31; 95% CrI: −1.90 to −0.69), with a minimum effective dose of 590 METs-min/week. For CT, MBE, and AE, the optimal doses were 1,000, 390, and 750 METs-min/week, respectively, with minimum effective doses of 670, 290, and 580 METs-min/week.

## 4 Discussion

This network meta-analysis and dose–response investigation demonstrated that various exercise modalities, including HIIT, CT, MBE, RT, and AE, effectively improve QoL in patients with HF. Higher total physical activity doses were associated with greater QoL improvement. However, response patterns varied by modality. HIIT and CT showed clear dose-dependent improvements, while MBE and RT exhibited a U-shaped association. In contrast, the effect of AE plateaued at moderate-to-high doses.

Previous studies have shown that several factors contribute to reduced QoL, including depressive mood, younger age, elevated BMI, more severe symptoms, reduced systolic pressure, gender, functional status, ethnicity, sleep apnea, diminished sense of control, and prognostic uncertainty (32, 33).

The dose-dependent improvements observed in HIIT and CT may be explained by multiple physiological mechanisms. HIIT generates greater shear stress forces within the endothelium, thereby enhancing endothelial function and improving oxygen metabolism (34, 35). CT, which combines aerobic and resistance exercises, offers both cardiovascular (enhanced aerobic performance) and musculoskeletal (increased muscular strength and functional performance) benefits (36). Compared with AE alone, CT significantly improves VO_2_ kinetics, submaximal exercise capacity, flow-mediated vasodilation, muscle strength, neuroendocrine activity, and inflammatory responses (37, 38).

In contrast, MBE (e.g., Tai Chi, Yoga, and Qigong) demonstrated a U-shaped dose–response curve, showing effectiveness even at low dosages. Previous studies have demonstrated that MBE yields multiple benefits, including improved sleep quality, reduced fatigue (39, 40), better balance, increased joint flexibility, and higher LVEF. It may also help alleviate symptoms associated with osteoarthritis and menopause, while improving cardiopulmonary function, blood lipid profiles, and hypertension (41, 42). Furthermore, MBE also improves cognitive function and reduces stress, anxiety, and depression (43).

RT and AE also demonstrated efficacy in improving QoL in patients with heart failure. Both modalities can improve peak VO_2_, exercise workload, cardiac performance, LVEF, and skeletal muscle mass and function. However, AE showed a smaller effect in our study, despite remaining the most widely used training modality to date (10, 44). A plateau in AE efficacy was observed at moderate-to-high doses. Regarding RT, the dose–response curve did not demonstrate statistical significance, which may be attributed to the limited number of studies investigating resistance exercise in isolation, as most protocols combine RT with AE.

The analysis suggests that improvements in QoL are driven by distinct mechanisms depending on the exercise modality. MBE (e.g., Tai Chi, Yoga, and Qigong) appears to exert its primary effects on the psychological dimension. It alleviates anxiety, depression, and sleep disorders, which are critical components of QoL that are less directly addressed by pure aerobic training (39, 40, 43). In comparison, HIIT, AE, and CT primarily enhance the physical dimension of QoL. These modalities directly target cardiovascular adaptations, such as enhanced cardiac output and improved aerobic capacity, which directly translate into a reduced symptom burden and improved physical functioning (34–37). RT and CT specifically target the functional domain by increasing skeletal muscle mass and strength, thereby preventing sarcopenia, which is often linked to physical dependency and prolonged hospital admissions. Such improvements are crucial for performing activities of daily living, contributing to a greater sense of independence and functional status (45).

From a clinical perspective, these findings support the use of personalized exercise prescriptions for patients with HF. Based on the dose–response analysis, patients with milder symptoms and higher exercise tolerance are ideal candidates for HIIT and CT. For these modalities, improvements in QoL increase progressively with increasing doses. Consequently, clinicians should aim to progressively increase training volume to maximize QoL benefits. To reach the minimum effective dose of 590 METs-min/week, a regimen of approximately 25 min of cycling-based HIIT performed three times per week is recommended.

For patients with severe symptoms, lower exercise tolerance, or anxiety, MBE represents a suitable intervention. Its gentle movements and low intensity ensure low risk and promote high adherence. The optimal dose is relatively low (390 METs-min/week), which corresponds to approximately 40 min of Tai Chi or Qigong performed three times per week. However, clinicians should be cautious not to overprescribe MBE volume, as the U-shaped curve suggests diminishing returns at higher doses.

While AE remains a foundational therapy, the plateau observed at higher doses suggests that simply increasing the duration or intensity of continuous aerobic exercise may not yield proportional gains in QoL. The minimum effective dose for AE is 580 METs-min/week, corresponding to approximately 40 min of low-to-moderate intensity stationary cycling performed three times per week. For patients already performing AE without further improvement, switching to HIIT or incorporating resistance training may help overcome this therapeutic plateau.

In summary, HIIT and CT may be more suitable for patients with milder HF, as these patients can tolerate and adapt to increased training loads. The therapeutic efficacy of these modalities increases with higher training volumes. Conversely, MBE may be ideal for patients with severe HF, as it offers clinical benefits at lower doses with low risk.

## 5 Limitations and future directions

Our study has several limitations. First, the 95% CrI for RT crossed the zero line, which may reflect the limited number of studies investigating RT as a standalone intervention , rather than a true lack of effect. We found only three studies that met our inclusion criteria and were suitable for dose–response analysis. Most trials combined RT with AE. Future studies should focus on RT in isolation.

Second, the estimation of exercise dose may be imprecise, despite the use of objective measures. We used weighted averages to estimate the progressively increasing intensities and utilized range midpoints to estimate dose ranges. These methodological approximations may have introduced uncertainty into the dose–response modeling.

Third, we included both per-protocol and intention-to-treat data, as well as different types of heart failure, due to the limited number of studies for certain exercise modalities. These limitations may have increased heterogeneity. Therefore, the results should be interpreted with caution.

Finally, although our meta-regression analysis suggested that treatment benefits were consistent regardless of mean age or baseline LVEF, we acknowledge the inherent physiological heterogeneity within the heart failure population. As many previous trials enrolled mixed cohorts or predated modern HFrEF/HFpEF classifications, our results reflect aggregate effects. Future trials specifically stratified by distinct HF phenotypes are warranted to further validate these findings.

## 6 Conclusion

This systematic review and network meta-analysis suggests that HIIT, CT, MBE, and RT demonstrate strong and comparable efficacy in improving QoL in patients with HF. In contrast, AE offers a more modest benefit. Notably, MBE appears effective even at relatively low doses, whereas CT and HIIT show progressive improvements with increasing doses.

## Data Availability

The raw data supporting the conclusions of this article will be made available by the authors, without undue reservation.

## Supplementary material

The Supplementary Materialfor this article can be found online at: https://www.frontiersin.org/articles/10.3389/fcvm.2026.1774345/full#supplementary-material
