# Supplementary material for: Comparative efficacy of various exercise types and doses for quality of life in patients with heart failure: a network and dose–response meta-analysis
Source: Front Cardiovasc Med. 2026 Mar 25;13:1774345. doi: 10.3389/fcvm.2026.1774345 (PMC13057295; doi:10.3389/fcvm.2026.1774345)

Supplementary Material

- **Supplemenatry 1.1 Search strategy**
- **Database: PubMed <inception to Mar 1 2025>**

| Search number | Query |
| --- | --- |
| 10 | ((("Exercise"[Mesh]) OR (Exercises or Exercise, Physical or Exercises, Physical or Physical Exercise or Physical Exercises or Exercise, Aerobic or Aerobic Exercise or Aerobic Exercises or Exercises, Aerobic or Exercise, Isometric or Exercises, Isometric or Isometric Exercises or Isometric Exercise or Acute Exercise or Acute Exercises or Exercise, Acute or Exercises, Acute or Exercise Training or Exercise Trainings or Training, Exercise or Trainings, Exercise or Physical Activity or Activities, Physical or Activity, Physical or Physical Activities)) AND (("Heart Failure"[Mesh]) OR (Cardiac Failure or Heart Decompensation or Decompensation, Heart or Congestive Heart Failure or Heart Failure, Congestive or Heart Failure, Right-Sided or Heart Failure, Right Sided or Right-Sided Heart Failure or Right Sided Heart Failure or Heart Failure, Left-Sided or Heart Failure, Left Sided or Left-Sided Heart Failure or Left Sided Heart Failure or Myocardial Failure))) AND (("Quality of Life"[Mesh]) OR (Life Quality or Health-Related Quality Of Life or Health Related Quality Of Life or HRQOL)) |
| 9 | ("Quality of Life"[Mesh]) OR (Life Quality or Health-Related Quality Of Life or Health Related Quality Of Life or HRQOL) |
| 8 | Life Quality or Health-Related Quality Of Life or Health Related Quality Of Life or HRQOL |
| 7 | "Quality of Life"[Mesh] |
| 6 | ("Heart Failure"[Mesh]) OR (Cardiac Failure or Heart Decompensation or Decompensation, Heart or Congestive Heart Failure or Heart Failure, Congestive or Heart Failure, Right-Sided or Heart Failure, Right Sided or Right-Sided Heart Failure or Right Sided Heart Failure or Heart Failure, Left-Sided or Heart Failure, Left Sided or Left-Sided Heart Failure or Left Sided Heart Failure or Myocardial Failure) |
| 5 | Cardiac Failure or Heart Decompensation or Decompensation, Heart or Congestive Heart Failure or Heart Failure, Congestive or Heart Failure, Right-Sided or Heart Failure, Right Sided or Right-Sided Heart Failure or Right Sided Heart Failure or Heart Failure, Left-Sided or Heart Failure, Left Sided or Left-Sided Heart Failure or Left Sided Heart Failure or Myocardial Failure |
| 4 | "Heart Failure"[Mesh] |
| 3 | ("Exercise"[Mesh]) OR (Exercises or Exercise, Physical or Exercises, Physical or Physical Exercise or Physical Exercises or Exercise, Aerobic or Aerobic Exercise or Aerobic Exercises or Exercises, Aerobic or Exercise, Isometric or Exercises, Isometric or Isometric Exercises or Isometric Exercise or Acute Exercise or Acute Exercises or Exercise, Acute or Exercises, Acute or Exercise Training or Exercise Trainings or Training, Exercise or Trainings, Exercise or Physical Activity or Activities, Physical or Activity, Physical or Physical Activities) |
| 2 | Exercises or Exercise, Physical or Exercises, Physical or Physical Exercise or Physical Exercises or Exercise, Aerobic or Aerobic Exercise or Aerobic Exercises or Exercises, Aerobic or Exercise, Isometric or Exercises, Isometric or Isometric Exercises or Isometric Exercise or Acute Exercise or Acute Exercises or Exercise, Acute or Exercises, Acute or Exercise Training or Exercise Trainings or Training, Exercise or Trainings, Exercise or Physical Activity or Activities, Physical or Activity, Physical or Physical Activities |
| 1 | "Exercise"[Mesh] |

- **Database: Ovid Medline <inception to Mar 1 2025>**

| Search number | Query |
| --- | --- |
| 1 | Exercise/ |
| 2 | (exercise or exercises or exercise, physical or exercises, physical or physicalphysical exercises or exercise, aerobic or aerobicaerobic exercises or exercises, aerobic or exercise, isometric or exercises, isometric or isometric exercises or isometricacuteacute exercises or exercise, acute or exercises, acute or exercise training or exercise trainings or training,trainings,physical activity or activities, physical or activity, physical or physical activities).ab,ti,kw. |
| 3 | 1 or 2 |
| 4 | Heart Failure/ |
| 5 | (heart failure or cardiac failure or heart decompensation or decompensation, heart or congestiveheart failure, congestive or heart failure, right-sided or heart failure, right sided or right-sidedright sidedheart failure, left-sided or heart failure, left sided or left-sidedleft sidedmyocardial failure).ab,ti,kw. |
| 6 | 4 or 5 |
| 7 | Quality of Life/ |
| 8 | (quality of life or life quality or health-relatedhealth relatedhrqol).ab,ti,kw. |
| 9 | 7 or 8 |
| 10 | 3 and 6 and 9 |

- **Database: Embase <inception to Mar 1 2025>**

| Search number | Query |
| --- | --- |
| 1 | 'exercise'/exp |
| 2 | 'biometric exercise':ti,ab,kw OR 'effort':ti,ab,kw OR 'exercise capacity':ti,ab,kw OR 'exercise performance':ti,ab,kw OR 'exercise training':ti,ab,kw OR 'exertion':ti,ab,kw OR 'fitness training':ti,ab,kw OR 'fitness workout':ti,ab,kw OR 'physical conditioning, human':ti,ab,kw OR 'physical effort':ti,ab,kw OR 'physical exercise':ti,ab,kw OR 'physical exertion':ti,ab,kw OR 'physicalwork-out':ti,ab,kw OR 'physical workout':ti,ab,kw OR 'exercise':ti,ab,kw |
| 3 | #1 OR #2 |
| 4 | 'heart failure'/exp |
| 5 | 'backward failure, heart':ti,ab,kw OR 'cardiac backward failure':ti,ab,kw OR 'cardiac decompensation':ti,ab,kw OR 'cardiac failure':ti,ab,kw OR 'cardiac incompetence':ti,ab,kw OR 'cardiac insufficiency':ti,ab,kw OR 'cardiac stand still':ti,ab,kw OR 'cardial decompensation':ti,ab,kw OR 'cardial insufficiency':ti,ab,kw OR 'chronic heart failure':ti,ab,kw OR 'chronic heart insufficiency':ti,ab,kw OR 'decompensatio cordis':ti,ab,kw OR 'decompensation, heart':ti,ab,kw OR 'heart backward failure':ti,ab,kw OR 'heart decompensation':ti,ab,kw OR 'heart  incompetence':ti,ab,kw OR 'heart insufficiency':ti,ab,kw OR 'insufficientia cardis':ti,ab,kw OR 'myocardial failure':ti,ab,kw OR 'myocardial insufficiency':ti,ab,kw OR 'heart failure':ti,ab,kw |
| 6 | #4 OR #5 |
| 7 | 'quality of life'/exp |
| 8 | 'health related quality of life':ti,ab,kw OR 'hrql':ti,ab,kw OR 'life quality':ti,ab,kw OR 'quality of life':ti,ab,kw |
| 9 | #7 OR #8 |
| 10 | #3 AND #6 AND #9 |

- **Database: Cochrane <inception to Mar 1 2025>**

ID Search

#1 MeSH descriptor: [Exercise] explode all trees

#2 (Activities, Physical or Physical Activities or Activity, Physical or Physical Activity or Exercises, Isometric or Exercise, Isometric or Isometric Exercises or Isometric Exercise or Exercise Trainings or Exercise Training or Trainings, Exercise or Training, Exercise or Exercise, Aerobic or Aerobic Exercise or Aerobic Exercises or Exercises, Aerobic or Acute Exercises or Acute Exercise or Exercise, Acute or Exercises, Acute or Physical Exercises or Exercise, Physical or Exercises, Physical or Exercises or Physical Exercise):ti,ab,kw (Word variations have been searched)

#3 #1 or #2

#4 MeSH descriptor: [Heart Failure] explode all trees

#5 (Heart Failure, Congestive or Congestive Heart Failure or Cardiac Failure or Right-Sided Heart Failure or Heart Failure, Right Sided or Heart Failure, Right-Sided or Right Sided Heart Failure or Heart Failure, Left Sided or Heart Failure, Left-Sided or Left-Sided Heart Failure or Left Sided Heart Failure or Myocardial Failure or Heart Decompensation or Decompensation, Heart):ti,ab,kw (Word variations have been searched)

#6 #4 or #5

#7 MeSH descriptor: [Quality of Life] explode all trees

#8 (HRQOL or Health Related Quality Of Life or Health-Related Quality Of Life or Life Quality):ti,ab,kw (Word variations have been searched)

#9 #7 or #8

#10 #3 and #6 and #9

- **Database: Web of science <inception to Mar 1 2025>**

| Search number | Query |
| --- | --- |
| 1 | (TS=(Exercise)) OR TS=(Exercises or Exercise, Physical or Exercises, Physical or Physical Exercise or Physical Exercises or Exercise, Aerobic or Aerobic Exercise or Aerobic Exercises or Exercises, Aerobic or Exercise, Isometric or Exercises, Isometric or Isometric Exercises or Isometric Exercise or Acute Exercise or Acute Exercises or Exercise, Acute or Exercises, Acute or Exercise Training or Exercise Trainings or Training, Exercise or Trainings, Exercise or Physical Activity or Activities, Physical or Activity, Physical or Physical Activities) |
| 2 | (TS=(Heart failure)) OR TS=(Cardiac Failure or Heart Decompensation or Decompensation, Heart or Congestive Heart Failure or Heart Failure, Congestive or Heart Failure, Right-Sided or Heart Failure, Right Sided or Right-Sided Heart Failure or Right Sided Heart Failure or Heart Failure, Left-Sided or Heart Failure, Left Sided or Left-Sided Heart Failure or Left Sided Heart Failure or Myocardial Failure) |
| 3 | (TS=(quality of life)) OR TS=(Life Quality or Health-Related Quality Of Life or Health Related Quality Of Life or HRQOL) |
| 4 | #1 and #2 and #3 |

- **Supplemenatry 1.2 Charactiristics of studies and subjects.**

| **Number** | **Author** | **Mean age  (EX / control)** | **Subjects  (EX/ control)** | **Sex (m/f) (EX / control)** | **Physical condition** | **Intervention detail (frequency, duration,sessions,time,intensity)** | **Adherence** | **OUTCOME** | **For dose reponse** | **Comorbidity** |
| --- | --- | --- | --- | --- | --- | --- | --- | --- | --- | --- |
| 1 | Beniaminovitz  2002 | CT:50(3) CON:48(4) | CT:17 CON:8 | CT:12/5 CON:6/2 | HFrEF | CT:3 times/week, 12 weeks,36 sessions, 30 min, 50% of peak VO2 CON:health education | NA | MLHFQ | N | NA |
| 2 | Yeh  2013 | TAICHI:68(11) AE:63(11) | TAICHI:8 AE:8 | TAICHI:4/4 AE:4/4 | HFpEF | TAICHI:2 times/week,12 weeks, 24 sessions, 60 min, 3 times/week in home, 35 min AE:2 times/week,12 weeks, 24 sessions, 60 min, low to moderate intensity, 3 times/week in home, 35 min | TAICHI:89% AE:88% | MLHFQ | Y | Coronary artery disease, Arrhythmia Hypertension, Diabetes , High cholesterol,Asthma , Anxiety, Depression, Arthritis, Cancer , Renal disease |
| 3 | Munch  2018 | AE:63(3) RT:59(3) | AE:14 RT:12 | AE:12/2 RT:10/2 | HFrEF | AE:3 times/week, 6 weeks, 18 sessions, 45 min(10min warm-up, 35 min cycling), 75% peak work load RT:3 times/week, 6 weeks, 18 sessions,25 min(10 min warm-up, 15 min RT), 25%-40% 1RM | NA | MLHFQ | Y | NA |
| 4 | Tyni-Lenne 2001 | RT:63(9) CON:62 (11) | RT:16 CON:8 | RT:8/8 CON:5/3 | HFrEF | RT:3 times/week, 8 weeks, 24 sessions, 60 min(15 min warm-up and cool-down), 13-16 Borg RPE Scale CON:usual care | NA | MLHFQ | Y | Coronary artery disease, Hypertension |
| 5 | Kitzman 2013 | AE:70(7) CON:70(7) | AE:32 CON:31 | AE:9/23 CON:6/25 | HFpEF | AE:3 times/week, 16 weeks, 48 sessions, 60 min(40 min AE，10 min warm-up and cool-down), 40-70%HRR CON:attention control | AE:88% | MLHFQ | Y | Hypertension, Diabetes Mellitus |
| 6 | Kitzman 2010 | AE:70(6) CON:69(5) | AE:26 CON:27 | AE:6/20 CON:7/20 | HFpEF | AE:3 times/week, 16 weeks, 48 sessions, 60 min(30-40 min AE,10 min warm-up and cool-down), 40-50%HRR(2 weeks)，60-70%HRR(14 weeks) CON:attention control | AE:88% | MLHFQ | Y | Hypertension, Diabetes Mellitus, Pulmonary edema |
| 7 | Edelmann 2011 | CT:64(8) CON:65(6) | CT:44 CON:20 | CT:20/24 CON:8/12 | HFpEF | CT:2 times/week,first 4 weeks, 8 sessions, 20-40 min, 50-60% peak Vo2 3 times/week，final 8 weeks, 24 sessions,20-40 min, 70% peak vo2,60-65%1RM(10min)(2 times/week) CON:maintain usual activities | NA | MLHFQ | Y | Hypertension, Diabetes Mellitus, Hyperlipidemia |
| 8 | Brubaker 2009 | AE:70.4(5.3) CON:69.9(6.3) | AE:30 CON:29 | AE:19/11 CON:20/9 | HFrEF | AE:3 times/week, 16 weeks, 48 sessions, 60 min(30-40 min training),40-50%HRR(2 weeks) 60-70%HRR(14 weeks) CON:attention control | AE:93% | MLHFQ | Y | Diabetes mellitus, Hypertension, Pulmonary edema |
| 9 | Gary 2007 | 68(12) | AE:13 CON:10 | all women | HFpEF | AE:3 times/week, 12 weeks, 36 sessions, 20-30 min, 40-60% HRR CON:health education | NA | MLHFQ | Y | Hypertension, Diabetes mellitus, Ischemic HF Etiology, Chronic Obstructive Pulmonary Disease, Arthritism, Depression , Anxiety |
| 10 | Mandic 2009 | AE:63 (11) CT:59 (11) CON:62 (13) | AE:14 CT:15 CON:13 | AE:11/3 CT:11/4  CON:10/3 | HFrEF | AE:3 times/weeks, 12 weeks, 36 sessions,30 min, 50-70%HRR CT:3 times/weeks, 12 weeks, 36 sessions,30 min, 50-70%HRR and 1-2 sets of 6 resistance training (10 min)50%-70% 1RM.  CON:usual care | 78±25% | MLHFQ | Y | Dyslipidaemia, Hypertension, Ischaemic Heart Failure Aetiology, Obesity,Diabetes |
| 11 | Patwala 2009 | 64.4 | AE:25 CON:25 | NA | HFrEF | AE:3 times/week,12 weeks,30 min, 80%-90% peak HR CON:usual care | NA | MLHFQ | Y | Atrial Fibrillation |
| 12 | Servantes 2011 | AE:51.76(9.83) CT:50.82(9.45) CON:53(8.19) | AE:17 CT:17 CON:11 | AE:8/9 CT:9/8 CON:6/5 | HFrEF | AE:3-4 times/week, 12 weeks, 40 sessions, 30-45 min, at anaerobic threshold CT:12 weeks, 40 sessions, 10min, 30–40%1RM, CON:health education | AE:98.5 ±13.7% CT:100.2 ±11.2% | MLHFQ | Y | Sleep Apnoea, Hypertension, Dyslipidaemia,Diabetes, Obesity |
| 13 | Berg-Emons 2004 | AE:58.6 (12.1) CON:58.6 (10.6) | AE:18 CON:16 | AE:12/6 CON:13/3 | HFrEF | AE:2 times/week, 12 weeks, 24 sessions, 60 min, 60%HRR CON:usual care | NA | MLHFQ | Y | Ischemic Heart Disease, Hypertension, Valvular Disease |
| 14 | Mueller 2021 | HIIT:70(7) AE:70(8) CON:69(10) | HIIT:58 AE:58 CON:60 | HIIT:17/41 AE:23/35 CON:19/41 | HFpEF | HIIT:3 times/week, 12 weeks, 36 sessions, 38 min(10 min warm-up, 16 min HIIT，9 min interval),80%-90%HRR AE:5 times/week, 12 weeks, 60 sessions,40 min, 35-50% HRR,  CON:health education | HIIT:80.4% AE:76.4% | KCCQ | Y | Hypertension, Coronary Artery Disease, Diabetes, Hyperlipidemia, Atrial fibrillation |
| 15 | Ulbrich 2016 | HIIT:53.15(7.0) AE:54.02(9.9) | HIIT:12 AE:10 | HIIT:12/0 AE:10/0 | HFrEF | HIIT:3 times/week, 12weeks,36 sessions, 15 min(95% of peak heart rate),12 min(70% of peak HR)  AE:3 times/week, 12weeks,36 sessions,30 min(75% of peak HR) | NA | MLHFQ | Y | Ischaemic Heart Failure Etiology, Hypertension, Coronary Artery Disease, Obesity, Diabetes, Dyslipidemia |
| 16 | Laoutaris 2020 | CT:67.5(6.71) AE:64.8(8.75) | CT:17 AE:18 | CT:16/1 AE:16/2 | HFrEF | CT:3 times/week, 12 weeks, 36 sessions, 60 min,60-80% peakHR 30 min+ 50%1RM 30min AE:3 times/week, 12 weeks, 36 sessions, 60 min,60-80% peakHR | NA | MLHFQ | Y | Ischaemic Cardiomyopathy, Dilated Cardiomyopathy |
| 17 | Dracup 2007 | CT:54.6(12.5)  CON:53.3(12.7) | CT:86 CON:87 | CT:60/26 CON:64/23 | HFrEF | CT:4 times/week(AE) 3 times/week(RT), 24 weeks, 96 sessions, 10-45 min, 40-60% peak heart rate(AE), 80% 1RM(RT) CON:usual care | NA | MLHFQ | N | Hypertension, Diabetes, Ischemic Etiology, Dyslipidemia |
| 18 | Keteyian 1999 | AE:55(12) CON:57(12) | AE:21 CON:22 | NA | HFrEF | AE:3 times/week, 24 weeks, 72 sessions, 33min , 50-80%HRR CON:usual care | NA | MLHFQ | Y | NA |
| 19 | Gary 2010 | 65.8(13.5) | AE:20 CON:17 | NA | HFrEF,HFmrEF,HFpEF | AE:3 times/week,12 week, 36 sessions, 60 min,moderate intensity CON:usual care | AE:82% CON:72% | MLHFQ | N | Myocardial infarction, Hypertension, Diabetes, Depression |
| 20 | Belardinelli 1999 | AE:56(7) CON:53(9) | AE:50 CON:49 | AE:45/5 CON:43/6 | HFrEF | AE:3 times/week, 8 weeks,24 sessions,60min(15-20min warm-up, 40 min cycling), 60%peak VO2 CON:usual care | AE:89% | MLHFQ | Y | Ischemic Etiology, Hypertension, Diabetes |
| 21 | Yeh 2004 | TAICHI:66(12) CON:61(14) | TAICHI:15 CON:15 | TAICHI:10/5 CON:9/6 | HFrEF | TAICHI:2 times/week, 12 weeks, 24 sessions, 60 min CON:usual care | NA | MLHFQ | Y | Ischemic Etiology, Hypertension, Diabetes, Valvular Heart Disease, Arrhythmia |
| 22 | PULLEN 2008 | Yoga:52.1(3.3) CON:50.5(12.8) | Yoga:9 CON:10 | Yoga:2/7 CON:7/3 | HFrEF | YOGA:5 times/week, 8 weeks, 16 supervised sessions,24 at-home sessions, 70 min (10 min warm-up,40 min Yoga, 20 min relaxation） CON:usual care | NA | MLHFQ | Y | Coronary artery disease, Hypertension, Diabetes |
| 23 | CHRYSOHOOU 2013 | HIIT:63(9) CON:56(11) | HIIT:33 CON:39 | HIIT:29/4 CON:28/11 | HFrEF | HIIT:3 times/week, 12 weeks, 36 sessions, 45 min, 80-100% peak WR CON:usual care | NA | MLHFQ | Y | Ischemic Etiology, Hypertension, Hyperlipidemia, Diabetes, Obesity |
| 24 | Jo´ nsdo´ttir 2005 | CT:68(6.6) CON:69(5.3) | CT:21 CON:22 | CT:16/5 CON:18/4 | HFrEF | CT:2 times/week, 20 weeks, 40 sessions, 10 min warm-up, 15 min AE,20 min RT, 5 min cool-down,50% peak work load,20-25%1RM CON:health education | NA | Icelandic quality of life questionnaire | Y | Ischemic Etiology, Hypertension, Atrial Fibrillation, Valvular disease |
| 25 | Kaltsatou 2014 | CT:67.1(7.2) CON:67.2(5.0) | CT:16 CON:17 | NA | HFmrEF | CT:3 times/week, 32 weeks,96 sessions, 60 min(10 min warm-up ,20-40 min AE, 20 min RT, 10 min relaxation), Borg scale 13-14 and 60–85%1RM  CON:usual care | CT:89.5% | SF-36 | Y | Coronary artery disease, Hypertension, Valvular heart disease, Arrhythmia |
| 26 | FEIEREISEN 2007 | AE:59.4(6.5) RT:57.9(5.8) CT:60.6(5.6) CON:55.5(7.5) | AE:15 RT:15 CT:15 CON:15 | AE:11/4 RT:14/1 CT:13/2 CON:13/2 | HFrEF | AE:3 times/week, 13 weeks, 40 sessions,45 min (5 min warm-up,40 min training),first 10 sessions(60% VO2peak),final 30 sessions(75% VO2peak) RT:3 times/week, 13 weeks, 40 sessions,45 min (5 min warm-up,40 min training),first 20 sessions(60%1RM),final 20 sessions(70%1RM) CT:3 times/week, 13 weeks, 40 sessions,45 min (5 min warm-up,40 min training),intensity equal to AE and RT group CON:usual care | NA | MLHFQ | Y | NA |
| 27 | Norman 2012 | CT:56.0 (2.7) CON:63.0 (3.4) | CT:20 CON:20 | CT:11/9 CON:12/8 | HFrEF | CT:3 times/week, 24 weeks,72 sessions, 30 min, 40-70 HRR% CON:health education | NA | KCCQ | Y | NA |
| 28 | Passino 2005 | AE:60(2) CON:61(2) | AE:44 CON:41 | AE:39/5 CON:35/6 | HFrEF | AE:3 times/week, 36 weeks, 108 sessions, 30 min, 60% peak VO2 CON:usual care | NA | MLHFQ | Y | Ischemic Heart Disease, Atrial Fibrillation |
| 29 | Chen 2016 | QIGONG:69.08 (13.48) CON:71.44 (13.65) | QIGONG:39 CON:41 | QIGONG:18/21 CON:24/17 | HFpEF | QIGONG:3 times/week, 12 weeks, 36 sessions, 35 min CON:usual care | NA | MLHFQ | Y | NA |
| 30 | Gary 2011 | CT:59(11) CON:61(10) | CT:12 CON:12 | CT:7/5 CON:5/7 | HFrEF | CT:2-3 times/week, 12 weeks,36 sessions, 30-60min, 50-70%HRR, 12 to 15 repetitions CON:wait list | CT:91% | MLHFQ | Y | Hypertension, Myocardial infarction, Diabetes, Depression, Dyslipidemia |

**Studies included**

1. Beniaminovitz A, Lang CC, LaManca J, Mancini DM. Selective low-level leg muscle training alleviates dyspnea in patients with heart failure. J Am Coll Cardiol. 2002 Nov 6;40(9):1602-8. doi: 10.1016/s0735-1097(02)02342-2. PMID: 12427412.
2. Yeh GY, Wood MJ, Wayne PM, Quilty MT, Stevenson LW, Davis RB, Phillips RS, Forman DE. Tai chi in patients with heart failure with preserved ejection fraction. Congest Heart Fail. 2013 Mar-Apr;19(2):77-84. doi: 10.1111/chf.12005. Epub 2012 Oct 12. PMID: 23057654; PMCID: PMC3546234.
3. Munch GW, Rosenmeier JB, Petersen M, Rinnov AR, Iepsen UW, Pedersen BK, Mortensen SP. Comparative Effectiveness of Low-Volume Time-Efficient Resistance Training Versus Endurance Training in Patients With Heart Failure. J Cardiopulm Rehabil Prev. 2018 May;38(3):175-181. doi: 10.1097/HCR.0000000000000304. PMID: 29351130.
4. Tyni-Lenné R, Dencker K, Gordon A, Jansson E, Sylvén C. Comprehensive local muscle training increases aerobic working capacity and quality of life and decreases neurohormonal activation in patients with chronic heart failure. Eur J Heart Fail. 2001 Jan;3(1):47-52. doi: 10.1016/s1388-9842(00)00087-8. PMID: 11163735.
5. Kitzman DW, Brubaker PH, Herrington DM, Morgan TM, Stewart KP, Hundley WG, Abdelhamed A, Haykowsky MJ. Effect of endurance exercise training on endothelial function and arterial stiffness in older patients with heart failure and preserved ejection fraction: a randomized, controlled, single-blind trial. J Am Coll Cardiol. 2013 Aug 13;62(7):584-92. doi: 10.1016/j.jacc.2013.04.033. Epub 2013 May 9. PMID: 23665370; PMCID: PMC3740089.
6. Kitzman DW, Brubaker PH, Morgan TM, Stewart KP, Little WC. Exercise training in older patients with heart failure and preserved ejection fraction: a randomized, controlled, single-blind trial. Circ Heart Fail. 2010 Nov;3(6):659-67. doi: 10.1161/CIRCHEARTFAILURE.110.958785. Epub 2010 Sep 17. PMID: 20852060; PMCID: PMC3065299.
7. Edelmann F, Gelbrich G, Düngen HD, Fröhling S, Wachter R, Stahrenberg R, Binder L, Töpper A, Lashki DJ, Schwarz S, Herrmann-Lingen C, Löffler M, Hasenfuss G, Halle M, Pieske B. Exercise training improves exercise capacity and diastolic function in patients with heart failure with preserved ejection fraction: results of the Ex-DHF (Exercise training in Diastolic Heart Failure) pilot study. J Am Coll Cardiol. 2011 Oct 18;58(17):1780-91. doi: 10.1016/j.jacc.2011.06.054. PMID: 21996391.
8. Brubaker PH, Moore JB, Stewart KP, Wesley DJ, Kitzman DW. Endurance exercise training in older patients with heart failure: results from a randomized, controlled, single-blind trial. J Am Geriatr Soc. 2009 Nov;57(11):1982-9. doi: 10.1111/j.1532-5415.2009.02499.x. PMID: 20121952; PMCID: PMC2950161.
9. Gary R, Lee SY. Physical function and quality of life in older women with diastolic heart failure: effects of a progressive walking program on sleep patterns. Prog Cardiovasc Nurs. 2007 Spring;22(2):72-80. doi: 10.1111/j.0889-7204.2007.05375.x. PMID: 17541316.
10. Mandic S, Tymchak W, Kim D, et al. Effects of aerobic or aerobic and resistance training on cardiorespiratory and skeletal muscle function in heart failure: a randomized controlled pilot trial. Clinical Rehabilitation. 2009;23(3):207-216. doi:[10.1177/0269215508095362](https://doi.org/10.1177/0269215508095362)
11. Patwala AY, Woods PR, Sharp L, Goldspink DF, Tan LB, Wright DJ. Maximizing patient benefit from cardiac resynchronization therapy with the addition of structured exercise training: a randomized controlled study. J Am Coll Cardiol. 2009 Jun 23;53(25):2332-9. doi: 10.1016/j.jacc.2009.02.063. PMID: 19539142.
12. Servantes DM, Pelcerman A, Salvetti XM, Salles AF, de Albuquerque PF, de Salles FC, Lopes C, de Mello MT, Almeida DR, Filho JA. Effects of home-based exercise training for patients with chronic heart failure and sleep apnoea: a randomized comparison of two different programmes. Clin Rehabil. 2012 Jan;26(1):45-57. doi: 10.1177/0269215511403941. Epub 2011 Sep 21. PMID: 21937519.
13. van den Berg-Emons R, Balk A, Bussmann H, Stam H. Does aerobic training lead to a more active lifestyle and improved quality of life in patients with chronic heart failure? Eur J Heart Fail. 2004 Jan;6(1):95-100. doi: 10.1016/j.ejheart.2003.10.005. PMID: 15012924.
14. Mueller S, Winzer EB, Duvinage A, Gevaert AB, Edelmann F, Haller B, Pieske-Kraigher E, Beckers P, Bobenko A, Hommel J, Van de Heyning CM, Esefeld K, von Korn P, Christle JW, Haykowsky MJ, Linke A, Wisløff U, Adams V, Pieske B, van Craenenbroeck EM, Halle M; OptimEx-Clin Study Group. Effect of High-Intensity Interval Training, Moderate Continuous Training, or Guideline-Based Physical Activity Advice on Peak Oxygen Consumption in Patients With Heart Failure With Preserved Ejection Fraction: A Randomized Clinical Trial. JAMA. 2021 Feb 9;325(6):542-551. doi: 10.1001/jama.2020.26812. PMID: 33560320; PMCID: PMC7873782.
15. Ulbrich A.Z., Angarten V.G., Schmitt Netto A., Sties S.W., Bundchen D.C., Mara L.S.D., Cornelissen V.A., Carvalho T.D. (2016). Comparative effects of high intensity interval training versus moderate intensity continuous training on quality of life in patients with heart failure: Study protocol for a randomized controlled trial. Clinical Trials and Regulatory Science in Cardiology, 13, 21-28. <http://dx.doi.org/10.1016/j.ctrsc.2015.11.005>
16. Laoutaris ID, Piotrowicz E, Kallistratos MS, Dritsas A, Dimaki N, Miliopoulos D, Andriopoulou M, Manolis AJ, Volterrani M, Piepoli MF, Coats AJS, Adamopoulos S; ARISTOS-HF trial (Aerobic, Resistance, InSpiratory Training OutcomeS in Heart Failure) Investigators. Combined aerobic/resistance/inspiratory muscle training as the 'optimum' exercise programme for patients with chronic heart failure: ARISTOS-HF randomized clinical trial. Eur J Prev Cardiol. 2021 Dec 29;28(15):1626-1635. doi: 10.1093/eurjpc/zwaa091. PMID: 33624071.
17. Dracup K, Evangelista LS, Hamilton MA, Erickson V, Hage A, Moriguchi J, Canary C, MacLellan WR, Fonarow GC. Effects of a home-based exercise program on clinical outcomes in heart failure. Am Heart J. 2007 Nov;154(5):877-83. doi: 10.1016/j.ahj.2007.07.019. Epub 2007 Sep 12. PMID: 17967593.
18. Keteyian SJ, Brawner CA, Schairer JR, Levine TB, Levine AB, Rogers FJ, Goldstein S. Effects of exercise training on chronotropic incompetence in patients with heart failure. Am Heart J. 1999 Aug;138(2 Pt 1):233-40. doi: 10.1016/s0002-8703(99)70106-7. PMID: 10426833.
19. Gary RA, Dunbar SB, Higgins MK, Musselman DL, Smith AL. Combined exercise and cognitive behavioral therapy improves outcomes in patients with heart failure. J Psychosom Res. 2010 Aug;69(2):119-31. doi: 10.1016/j.jpsychores.2010.01.013. Epub 2010 Mar 12. PMID: 20624510; PMCID: PMC4143390.
20. Belardinelli R, Georgiou D, Cianci G, Purcaro A. Randomized, controlled trial of long-term moderate exercise training in chronic heart failure: effects on functional capacity, quality of life, and clinical outcome. Circulation. 1999 Mar 9;99(9):1173-82. doi: 10.1161/01.cir.99.9.1173. PMID: 10069785.
21. Yeh GY, Wood MJ, Lorell BH, Stevenson LW, Eisenberg DM, Wayne PM, Goldberger AL, Davis RB, Phillips RS. Effects of tai chi mind-body movement therapy on functional status and exercise capacity in patients with chronic heart failure: a randomized controlled trial. Am J Med. 2004 Oct 15;117(8):541-8. doi: 10.1016/j.amjmed.2004.04.016. PMID: 15465501.
22. Pullen PR, Nagamia SH, Mehta PK, Thompson WR, Benardot D, Hammoud R, Parrott JM, Sola S, Khan BV. Effects of yoga on inflammation and exercise capacity in patients with chronic heart failure. J Card Fail. 2008 Jun;14(5):407-13. doi: 10.1016/j.cardfail.2007.12.007. Epub 2008 May 27. PMID: 18514933.
23. Chrysohoou C, Tsitsinakis G, Vogiatzis I, Cherouveim E, Antoniou C, Tsiantilas A, Tsiachris D, Dimopoulos D, Panagiotakos DB, Pitsavos C, Koulouris NG, Stefanadis C. High intensity, interval exercise improves quality of life of patients with chronic heart failure: a randomized controlled trial. QJM. 2014 Jan;107(1):25-32. doi: 10.1093/qjmed/hct194. Epub 2013 Sep 30. PMID: 24082155.
24. Jonsdottir S, Andersen KK, Sigurosson AF, Sigurosson SB. The effect of physical training in chronic heart failure. Eur J Heart Fail. . 2006;8(1):97-101. doi:10.1016/j.ejheart.2005.05.002
25. Kaltsatou AC, Kouidi EI, Anifanti MA, Douka SI, Deligiannis AP. Functional and psychosocial effects of either a traditional dancing or a formal exercising training program in patients with chronic heart failure: a comparative randomized controlled study. Clin Rehabil. 2014 Feb;28(2):128-38. doi: 10.1177/0269215513492988. Epub 2013 Jul 17. PMID: 23864515.
26. Feiereisen P, Delagardelle C, Vaillant M, Lasar Y, Beissel J. Is strength training the more efficient training modality in chronic heart failure? Med Sci Sports Exerc. 2007 Nov;39(11):1910-7. doi: 10.1249/mss.0b013e31814fb545. PMID: 17986897.
27. Norman JF, Pozehl BJ, Duncan KA, Hertzog MA, Krueger SK. Effects of Exercise Training versus Attention on Plasma B-type Natriuretic Peptide, 6-Minute Walk Test and Quality of Life in Individuals with Heart Failure. Cardiopulm Phys Ther J. 2012 Dec;23(4):19-25. PMID: 23304096; PMCID: PMC3537186.
28. Passino C, Severino S, Poletti R, Piepoli MF, Mammini C, Clerico A, Gabutti A, Nassi G, Emdin M. Aerobic training decreases B-type natriuretic peptide expression and adrenergic activation in patients with heart failure. J Am Coll Cardiol. 2006 May 2;47(9):1835-9. doi: 10.1016/j.jacc.2005.12.050. Epub 2006 Apr 19. PMID: 16682309.
29. Chen DM, Yu WC, Hung HF, Tsai JC, Wu HY, Chiou AF. The effects of Baduanjin exercise on fatigue and quality of life in patients with heart failure: A randomized controlled trial. Eur J Cardiovasc Nurs. 2018 Jun;17(5):456-466. doi: 10.1177/1474515117744770. Epub 2017 Nov 30. PMID: 29189045.
30. Gary RA, Cress ME, Higgins MK, Smith AL, Dunbar SB. Combined aerobic and resistance exercise program improves task performance in patients with heart failure. Arch Phys Med Rehabil. 2011 Sep;92(9):1371-81. doi: 10.1016/j.apmr.2011.02.022. PMID: 21878207; PMCID: PMC4143394.

- **Supplmentary 1.3 Risk of Bias**
- **For ITT**


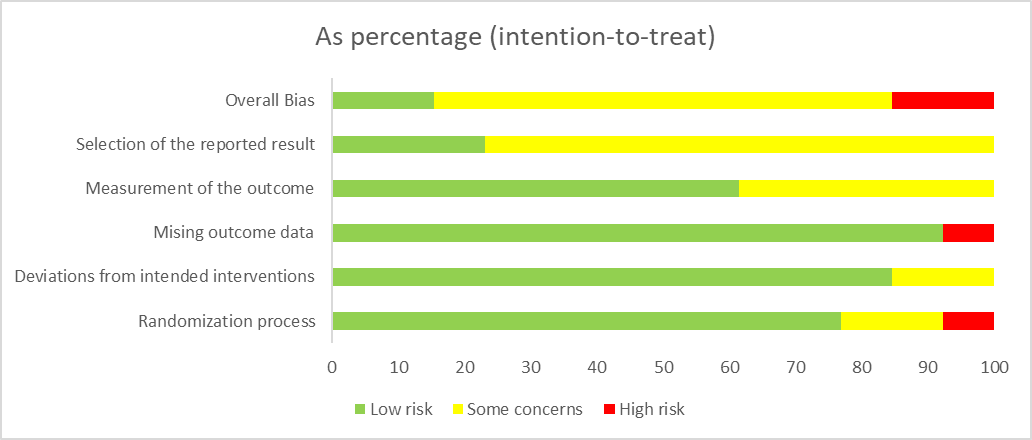


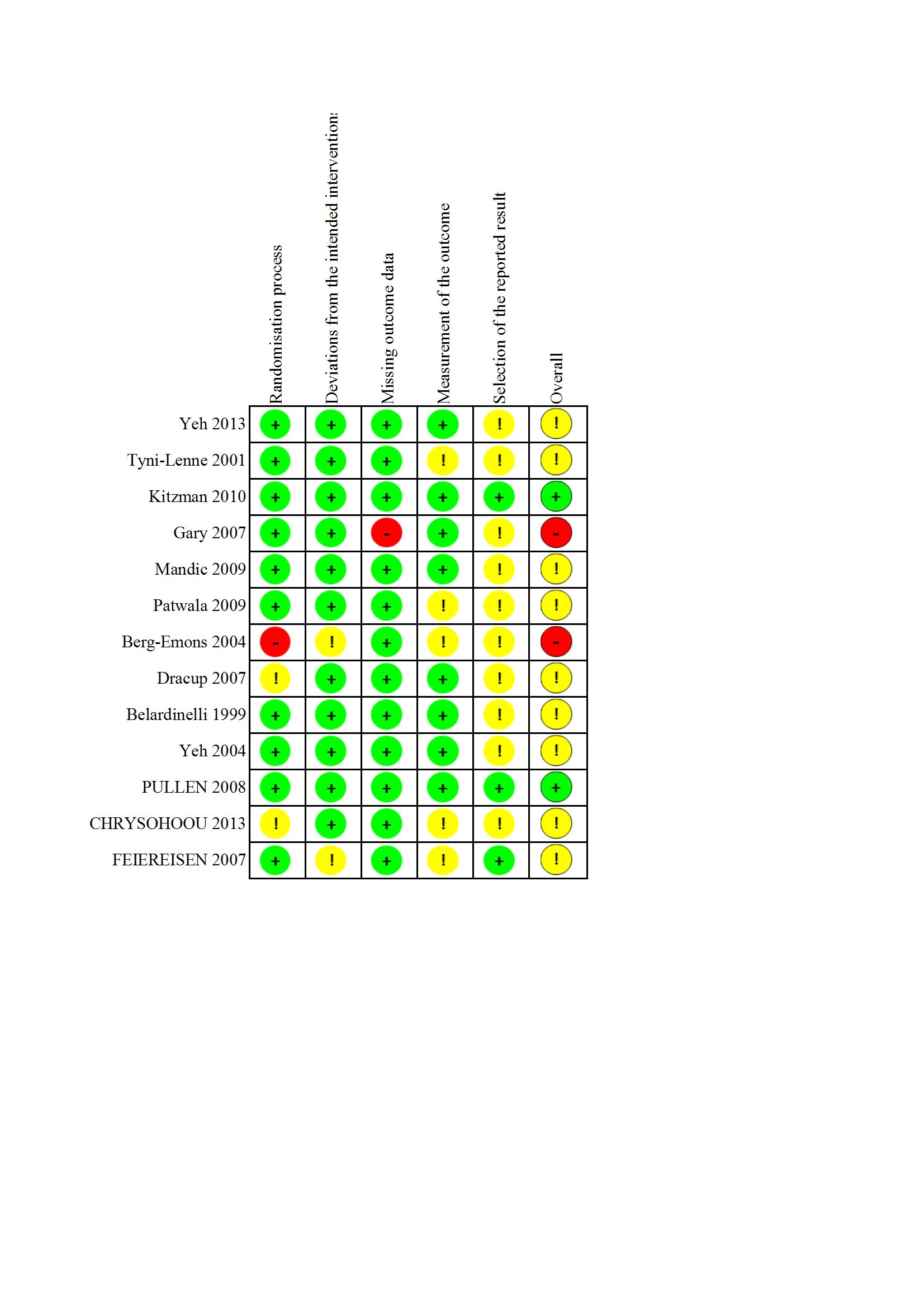


- **For PP**


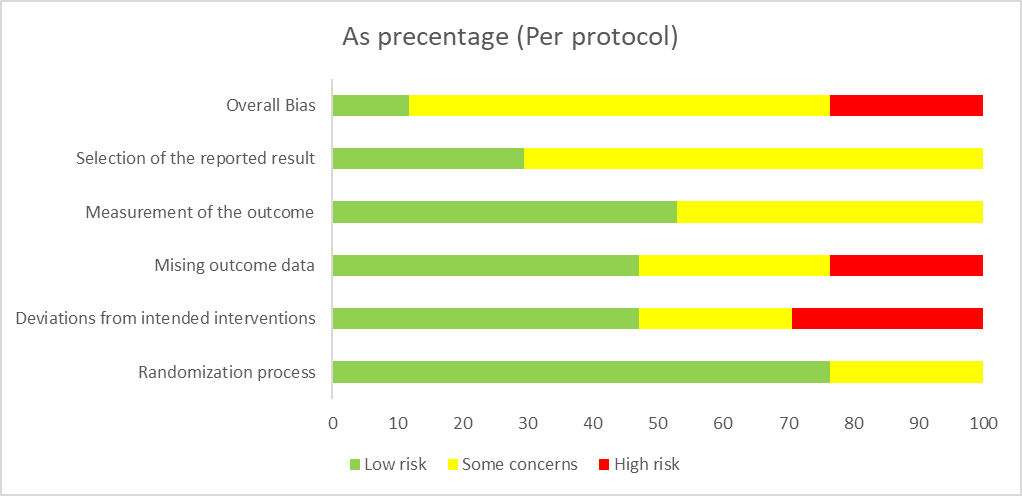

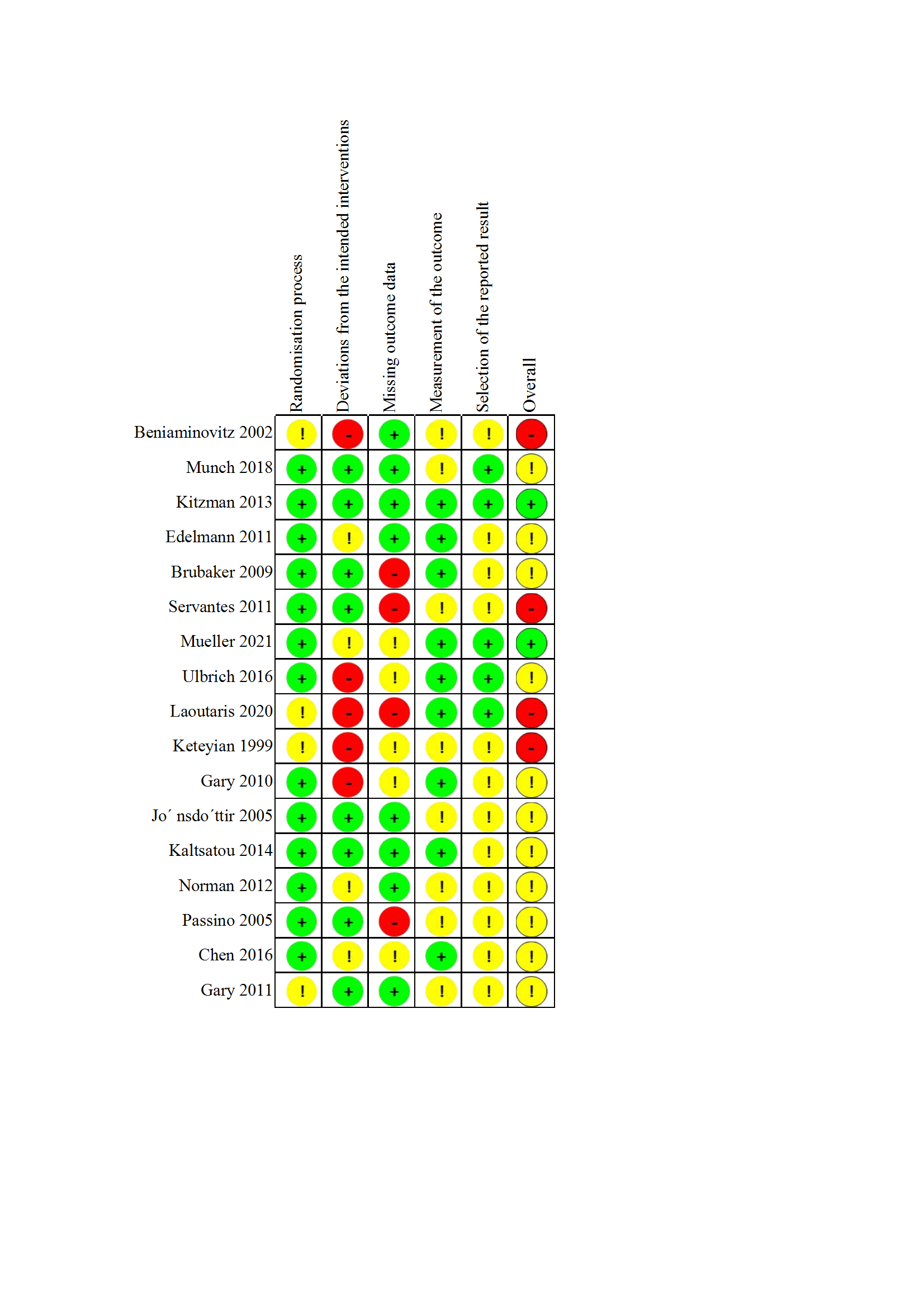


- **Supplmentary 1.4 Grading the evidence of the network meta-analysis using CINeMA**

**
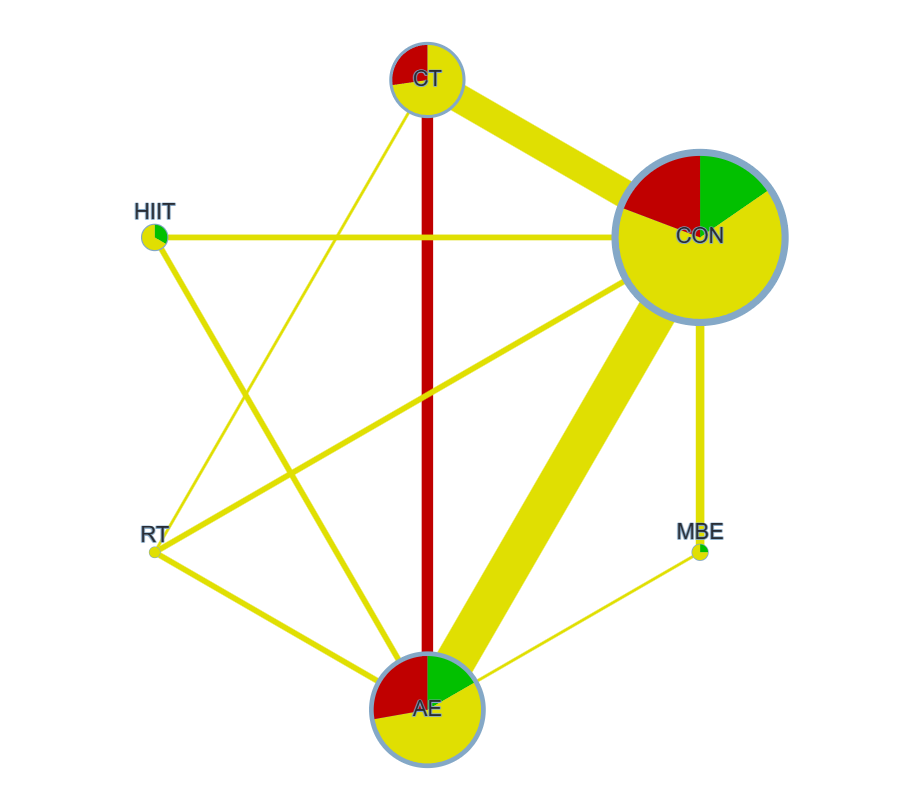
**

Network plot of study limitations of the included studies. Node size by sample size, node color by RoB. The colors in the circles indicate the percentage of low RoB studies (green), moderate RoB studies (yellow), high RoB studies (red) about each physical activity type. Edge width by number of studies. Edge color by average RoB. The colors of the lines indicate the summative RoB assessment of each comparison. Low RoB is green, moderate RoB is yellow, high RoB is red

**Reasons for downgrading**

Based on the recommendations of the CINeMA online document (https://cinema.ispm.unibe.ch/) , we judged whether each module needed to be downgraded according to the following criteria.

**With-study bias**

We classified the quality evaluation results of each included study into low-risk, moderate-risk and high-risk. We selected the rule is average RoB. No need to downgrade when the result was “no concerns”, downgrade one level when “some concerns” and downgrade two levels to “major concerns”.

**Across-study bias (publication bias)**

we evaluated the outcome of publication bias, and comparison-adjusted funnel plots showed no evidence of asymmetry. Additionally, Egger’s test (p=0.11) confirmed that there was no significant evidence for publication bias. Therefore, the outcome was deemed to have no publication bias.

**Indirectness**

We performed a point inconsistency test on the outcome and found no significant differences. Therefore, no indirectness was assumed and no comparison was downgraded for this reason.

**Imprecision**

The outcome of this network meta-analysis is a continuous variable, and the effect size measure for continuous outcomes chooses the standardized mean difference (SMD) of the change score (end-point minus baseline score) because the studies use different rating scales. Therefore, for CON comparisons the clinically meaningful threshold was set at a standardized mean difference of higher or lower than 0, and for the comparisons of the two types of exercise, the threshold was set at SMD -0.1 and 0.1. If the confidence interval crossed one threshold, it will be downgraded by one level, and two thresholds will be downgraded by two levels.

**Heterogeneity**

For heterogeneity, we used the same threshold as the above clinically significant threshold and follow the recommendations automatically provided by CINeMA (https://cinema.ispm.unibe.ch/). No need to downgrade when the result was “no concerns”, downgrade one level when “some concerns” and downgrade two level when “major concerns”.

**Incoherence**

For incoherence, we used global and local methods to test the inconsistency of the research results. For global inconsistency, we evaluated inconsistency statistically using the design-by-treatment test. In addition, we conducted assessment of local inconsistency by separating indirect from direct evidence (SIDE test) using the R netmeta package. No need to downgrade when p >0.1, downgrade one level when p was 0.05-0.1 and downgrade two levels when p <0.05.

**Summarizing judgments across the 6 domains**

The final output of CINeMA is a table with the level of concern for each of the 6 domains. we choose to summarise judgments across domains using the 4 levels of confidence of the GRADE approach: very low, low, moderate, or high. Due to factors that may reduce the confidence in a treatment effect may affect more than 1 domain. Indirectness includes consideration of intransitivity, which is manifested as statistical incoherence in the data. Heterogeneity will increase the imprecision of treatment effect, and may be related to the variability of bias within the study or the existence of reporting bias. In addition, in the presence of heterogeneity, the ability to detect important discontinuities will be reduced.Therefore, the 6 CINeMA domains should be considered jointly rather than in isolation to avoid downgrading the overall level of confidence more than once for related concerns.

- **CINeMA for the primary outcome**

| Comparison | Number of studies | Within-study bias | Reporting bias | Indirectness | Imprecision | Heterogeneity | Incoherence | Confidence rating | Reason(s) for downgrading |
| --- | --- | --- | --- | --- | --- | --- | --- | --- | --- |
| AE:CON | 14 | Some concerns | Low risk | No concerns | No concerns | Some concerns | No concerns | Low | ["Within-study bias","Heterogeneity"] |
| AE:CT | 4 | Some concerns | Low risk | No concerns | Major concerns | No concerns | No concerns | Very low | ["Within-study bias","Imprecision"] |
| AE:HIIT | 2 | Some concerns | Low risk | No concerns | Major concerns | No concerns | No concerns | Very low | ["Within-study bias","Imprecision"] |
| AE:MBE | 1 | Some concerns | Low risk | No concerns | Major concerns | No concerns | No concerns | Very low | ["Within-study bias","Imprecision"] |
| AE:RT | 2 | Some concerns | Low risk | No concerns | Major concerns | No concerns | No concerns | Very low | ["Within-study bias","Imprecision"] |
| CON:CT | 10 | Some concerns | Low risk | No concerns | No concerns | Some concerns | No concerns | Low | ["Within-study bias","Heterogeneity"] |
| CON:HIIT | 2 | Some concerns | Low risk | No concerns | No concerns | Major concerns | No concerns | Very low | ["Within-study bias","Heterogeneity"] |
| CON:MBE | 3 | Some concerns | Low risk | No concerns | No concerns | Major concerns | No concerns | Very low | ["Within-study bias","Heterogeneity"] |
| CON:RT | 2 | Some concerns | Low risk | No concerns | No concerns | Major concerns | No concerns | Very low | ["Within-study bias","Heterogeneity"] |
| CT:RT | 1 | Some concerns | Low risk | No concerns | Major concerns | No concerns | No concerns | Very low | ["Within-study bias","Imprecision"] |
| CT:HIIT | 0 | Some concerns | Low risk | No concerns | Major concerns | No concerns | No concerns | Very low | ["Within-study bias","Imprecision"] |
| CT:MBE | 0 | Some concerns | Low risk | No concerns | Major concerns | No concerns | No concerns | Very low | ["Within-study bias","Imprecision"] |
| HIIT:MBE | 0 | Some concerns | Low risk | No concerns | Major concerns | No concerns | No concerns | Very low | ["Within-study bias","Imprecision"] |
| HIIT:RT | 0 | Some concerns | Low risk | No concerns | Major concerns | No concerns | No concerns | Very low | ["Within-study bias","Imprecision"] |
| MBE:RT | 0 | Some concerns | Low risk | No concerns | Major concerns | No concerns | No concerns | Very low | ["Within-study bias","Imprecision"] |

- **Supplmentary 1.5 Classification and definition of exercise modalities.**

| Abbreviation | Full name | Definition |
| --- | --- | --- |
| HIIT | High-intensity interval training | High-intensity interval training (HIIT) involves alternating 3-4 min periods of exercise at 80%–90% HRR with exercise at 60%–70% HRR.[1] |
| MBE | Mind-body exercise | A form of exercise that combines body movement, mental focus, and controlled breathing to improve strength, balance, flexibility, and overall health. Examples of mind-body exercises are yoga, tai chi, and qigong[2, 3] |
| AE | Aerobic exercise | Continuous exercise at 40-80%Vo2 peak for 20-60 min[4] |
| RT | Resistance training | Resistance exercise including free weights, machines with stacked weights or pneumatic resistance, and resistance bands. Resistance training regimens focus on single-joint, multijoint or compound exercises that affect more than one muscle group.[1] |
| CT | Combined aerobic and resistance training | A combination of aerobic exercise and resistance training[1,5] |

1. American College of Sports Medicine., Liguori, G., Feito, Y., Fountaine, C., & Roy, B. (2022).ACSM's guidelines for exercise testing and prescription.Eleventh edition. Wolters Kluwer.
2. National Cancer Institute. (n.d.). Mind-body exercise. In NCI Dictionary of Cancer Terms. Retrieved Month Day, Year, from [https://www.cancer.gov/publications/dictionaries/cancer-terms/def/mind-body-exercise](https://www.cancer.gov/publications/dictionaries/cancer-terms/def/mind-body-exercise?utm_source=chatgpt.com)
3. National Center for Complementary and Integrative Health (NCCIH). (n.d.). Mind and body practices. U.S. Department of Health and Human Services. Retrieved Month Day, Year, from [https://www.nccih.nih.gov/health/mind-and-body-practices](https://www.nccih.nih.gov/health/mind-and-body-practices?utm_source=chatgpt.com)
4. Pelliccia A, Sharma S, Gati S, Bäck M, Börjesson M, Caselli S, Collet JP, Corrado D, Drezner JA, Halle M, Hansen D, Heidbuchel H, Myers J, Niebauer J, Papadakis M, Piepoli MF, Prescott E, Roos-Hesselink JW, Graham Stuart A, Taylor RS, Thompson PD, Tiberi M, Vanhees L, Wilhelm M; ESC Scientific Document Group. 2020 ESC Guidelines on sports cardiology and exercise in patients with cardiovascular disease. Eur Heart J. 2021 Jan 1;42(1):17-96. doi: 10.1093/eurheartj/ehaa605. Erratum in: Eur Heart J. 2021 Feb 1;42(5):548-549. doi: 10.1093/eurheartj/ehaa835. PMID: 32860412.
5. Shoemaker MJ, Dias KJ, Lefebvre KM, Heick JD, Collins SM. Physical Therapist Clinical Practice Guideline for the Management of Individuals With Heart Failure. Phys Ther. 2020 Jan 23;100(1):14-43. doi: 10.1093/ptj/pzz127. PMID: 31972027.

- **Supplmentary 2.1 Dose level network plot**


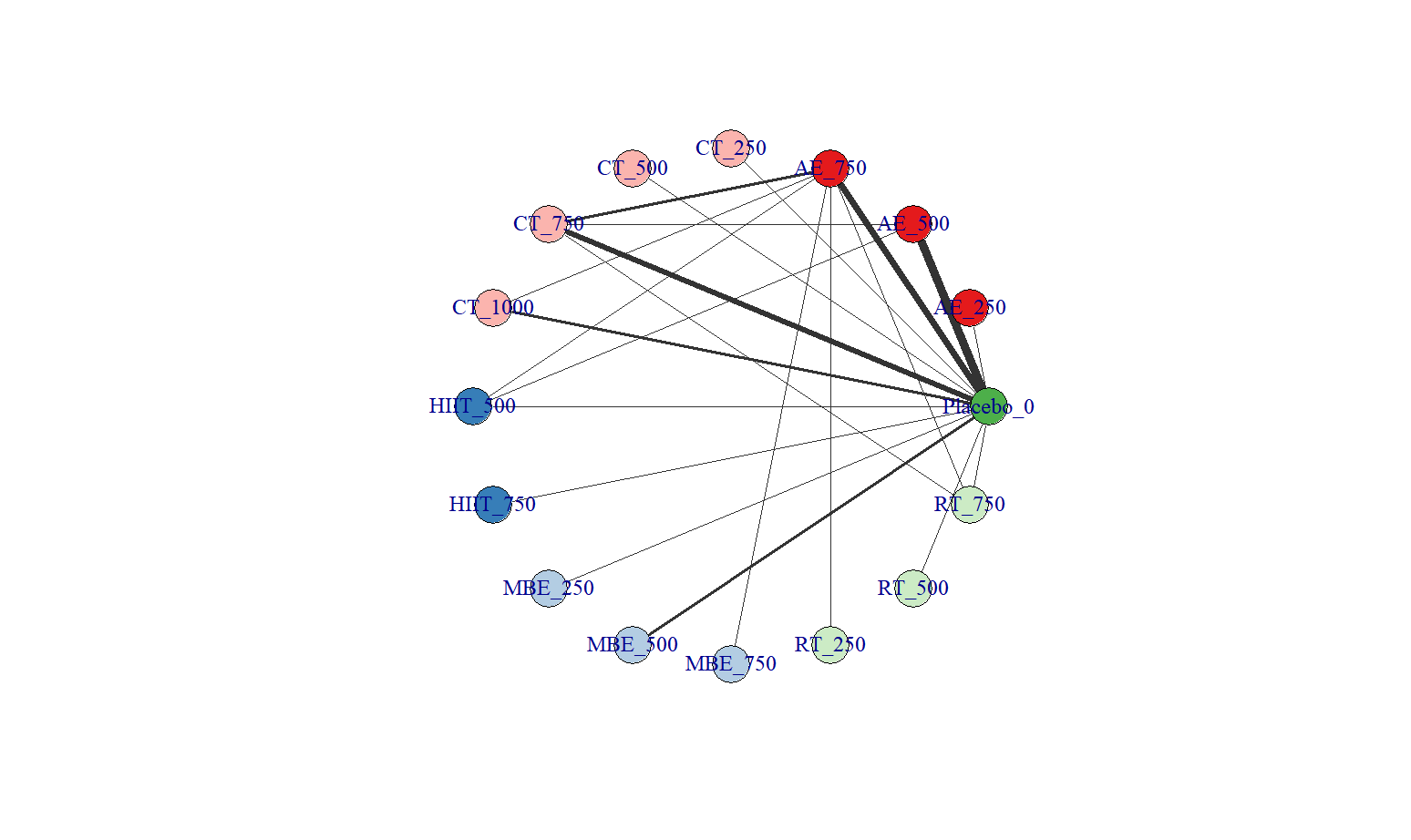


- **Supplementary 2.2 Treatment level network plot**


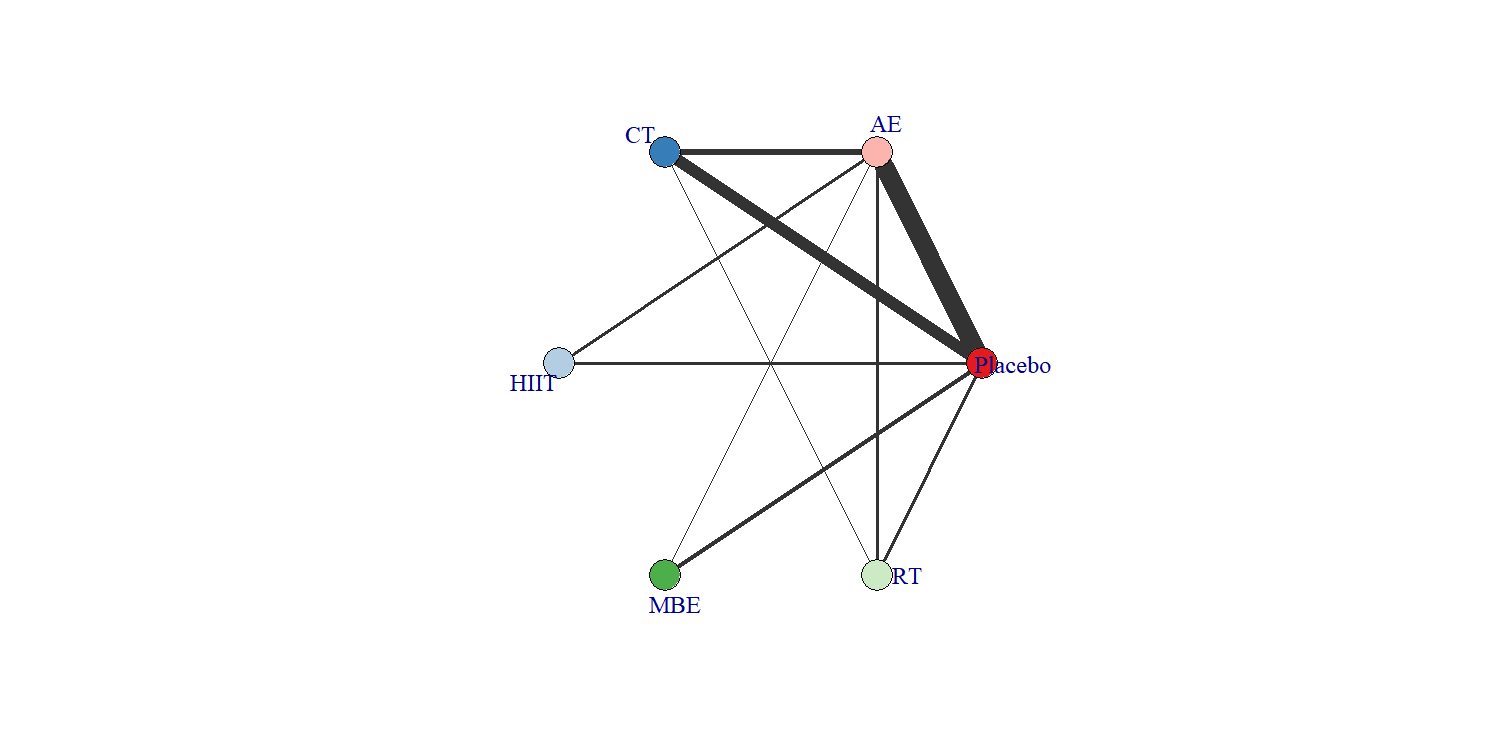


**Supplementary 2.3 “Split” NMA of different exercise agents**


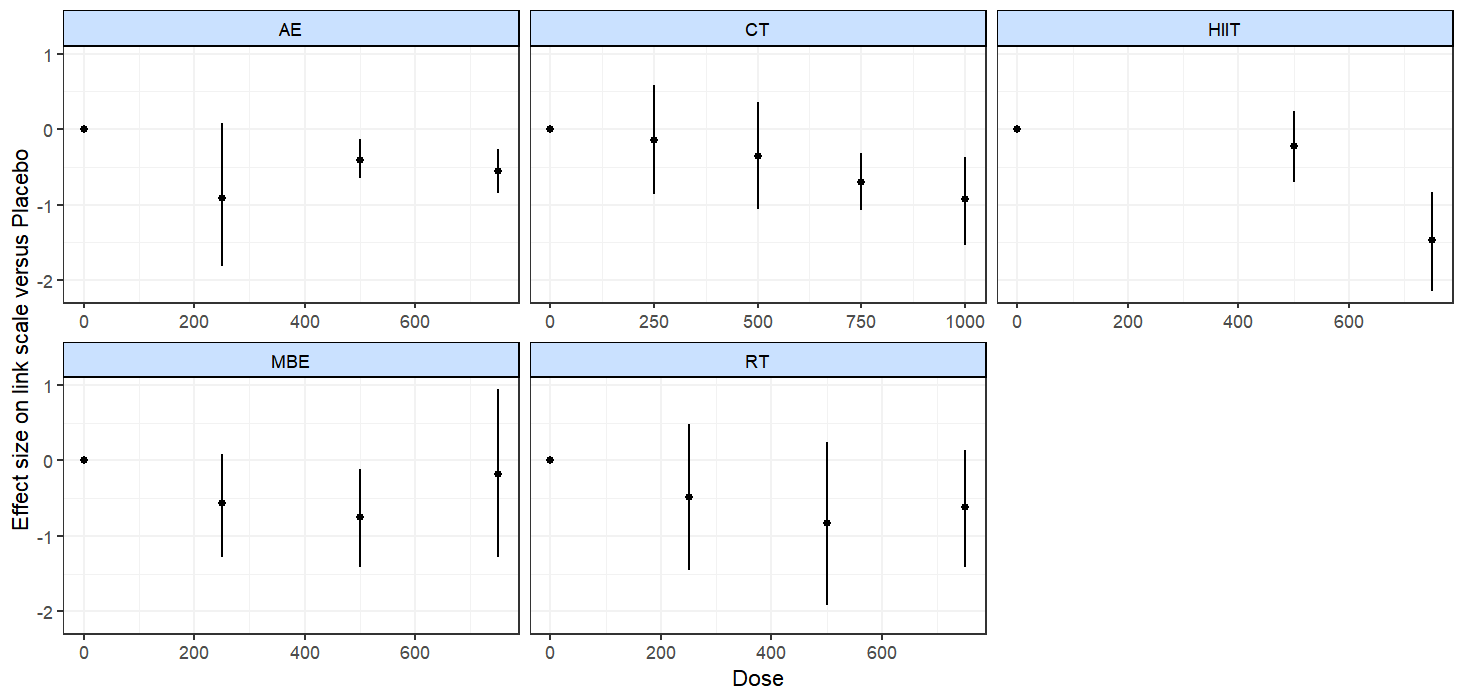


- **Supplementary 2.4 Comparison of different models**

| Model | Type | DIC | SD | Deviance | Residual Deviance | pD |
| --- | --- | --- | --- | --- | --- | --- |
| Emax | Common | 374.2 | NA | 342.086 | 82.709 | 31.9 |
| Emax | Random | 362.8 | 0.301 | 317.406 | 58.029 | 45.2 |
| Linear | Common | 367.2 | NA | 334.798 | 75.42 | 32.1 |
| Linear | Random | 361.2 | 0.246 | 318.711 | 59.334 | 43.3 |
| Exponential | Common | 376.4 | NA | 344.91 | 85.532 | 31.8 |
| Exponential | Random | 363.5 | 0.315 | 317.893 | 58.515 | 45.9 |
| Restricted cubic spline | Common | 361.1 | NA | 323.62 | 64.246 | 37.4 |
| Restricted cubic spline | Random | 359.8 | 0.17 | 318.22 | 58.842 | 41.6 |
| Non-parametric monotonically up | Common | 487.2 | NA | 457.974 | 198.596 | 28.4 |
| Non-parametric monotonically up | Random | 375.2 | 1.171 | 318.692 | 59.314 | 56.4 |

- **Supplementary 2.5 Deviance plot for each intervention and dose.**


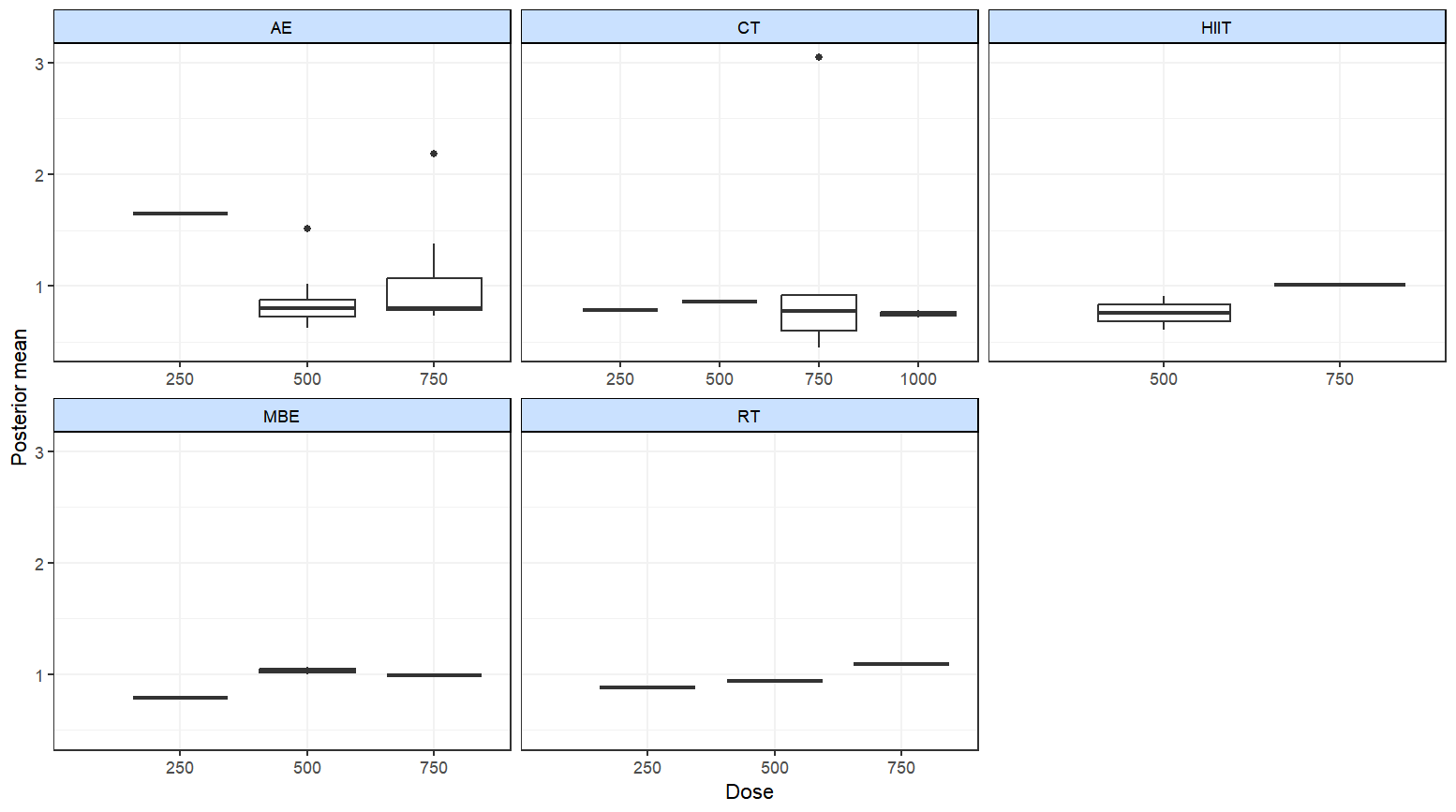


- **Supplementary 2.6 Consistent and UME models fit comparison**

| **Model** | **pD** | **Residual deviance** | **Deviance** | **DIC** | **SD** |
| --- | --- | --- | --- | --- | --- |
| Consistent | 43.3 | 59.1 | 318.5 | 362 | 0.25 |
| UME | 46.6 | 59.5 | 318.9 | 365.1 | 0.25 |

- **Supplementary 2.7 Posterior deviance distributions**


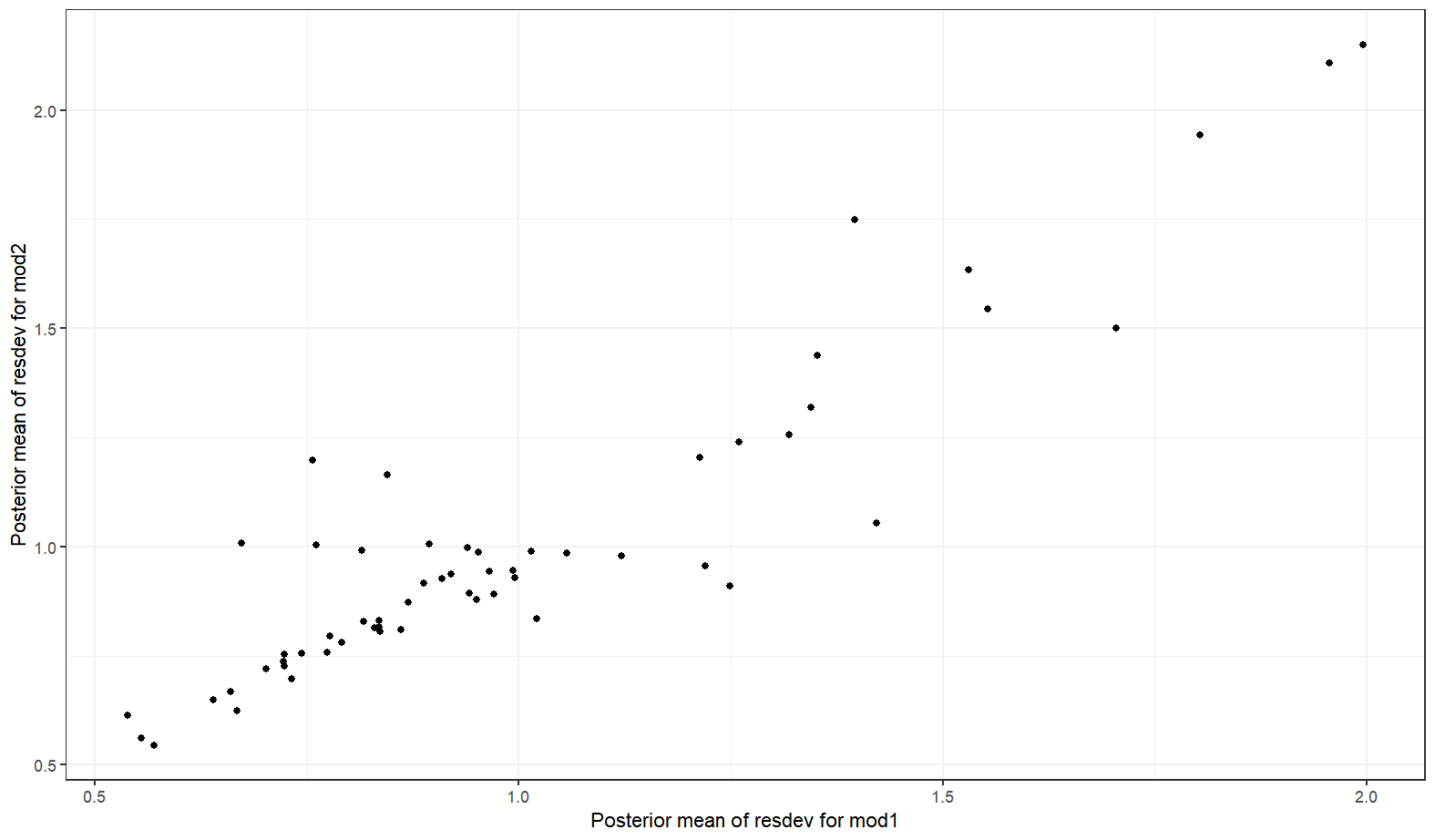


- **Supplementary 2.8 Density plots of the split comparisons**


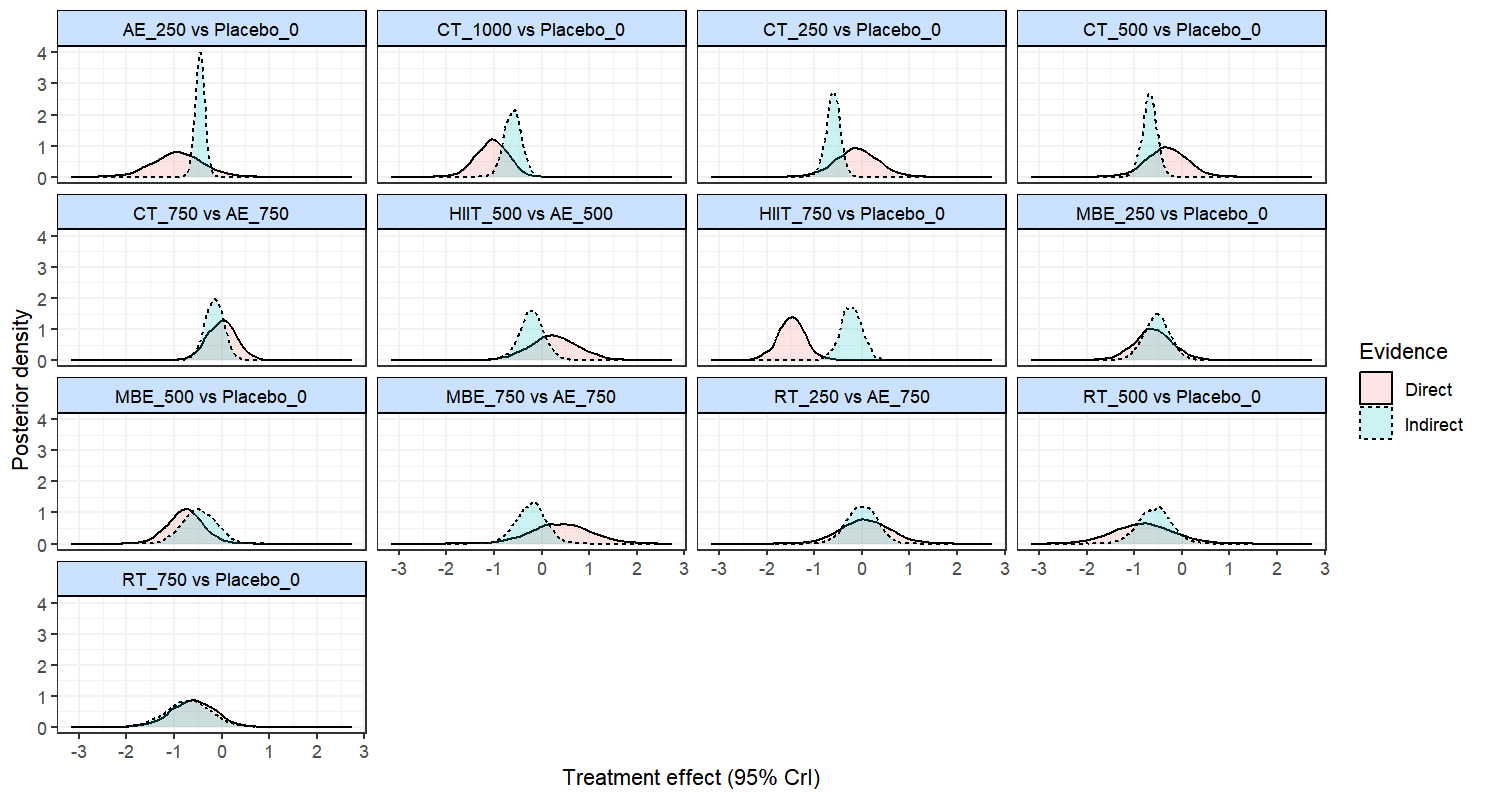


- **Supplementary 2.9 Details for MBNMA Node-splitting**

| Comparison | P-value | Median | 2.50% | 97.50% |
| --- | --- | --- | --- | --- |
| RT_250 vs AE_750 | 0.814 |  |  |  |
| -> direct |  | 0.084 | -0.814 | 0.905 |
| -> indirect |  | -0.069 | -1.292 | 1.099 |
| -> MBNMA |  | 0.006 | -0.671 | 0.689 |
|  |  |  |  |  |
| MBE_750 vs AE_750 | 0.556 |  |  |  |
| -> direct |  | 0.324 | -0.636 | 1.387 |
| -> indirect |  | -0.028 | -2.625 | 2.484 |
| -> MBNMA |  | 0.355 | -0.632 | 1.334 |
|  |  |  |  |  |
| CT_750 vs AE_750 | 0.704 |  |  |  |
| -> direct |  | -0.016 | -0.527 | 0.495 |
| -> indirect |  | -0.184 | -0.587 | 0.238 |
| -> MBNMA |  | -0.133 | -0.448 | 0.194 |
|  |  |  |  |  |
| HIIT_500 vs AE_500 | 0.759 |  |  |  |
| -> direct |  | 0.234 | -0.666 | 1.137 |
| -> indirect |  | 0.2 | -0.372 | 0.763 |
| -> MBNMA |  | 0.224 | -0.274 | 0.701 |
|  |  |  |  |  |
| RT_750 vs Placebo_0 | 0.345 |  |  |  |
| -> direct |  | -0.57 | -1.385 | 0.236 |
| -> indirect |  | -0.855 | -5.104 | 3.079 |
| -> MBNMA |  | -0.63 | -1.364 | 0.13 |
|  |  |  |  |  |
| RT_500 vs Placebo_0 | 0.863 |  |  |  |
| -> direct |  | -0.765 | -1.852 | 0.308 |
| -> indirect |  | -0.609 | -1.598 | 0.29 |
| -> MBNMA |  | -0.731 | -1.422 | -0.038 |
|  |  |  |  |  |
| MBE_500 vs Placebo_0 | 0.815 |  |  |  |
| -> direct |  | -0.791 | -1.38 | -0.162 |
| -> indirect |  | -0.637 | -1.417 | 0.065 |
| -> MBNMA |  | -0.74 | -1.212 | -0.244 |
|  |  |  |  |  |
| MBE_250 vs Placebo_0 | 0.857 |  |  |  |
| -> direct |  | -0.608 | -1.289 | 0.064 |
| -> indirect |  | -0.726 | -1.45 | -0.021 |
| -> MBNMA |  | -0.675 | -1.149 | -0.192 |
|  |  |  |  |  |
| HIIT_750 vs Placebo_0 | 0.014 |  |  |  |
| -> direct |  | -1.483 | -2.047 | -0.912 |
| -> indirect |  | -0.759 | -153.407 | 153.236 |
| -> MBNMA |  | -1.485 | -2.081 | -0.876 |
|  |  |  |  |  |
| CT_1000 vs Placebo_0 | 0.645 |  |  |  |
| -> direct |  | -0.978 | -1.587 | -0.428 |
| -> indirect |  | -1.137 | -2.427 | 0.02 |
| -> MBNMA |  | -0.977 | -1.498 | -0.509 |
|  |  |  |  |  |
| CT_500 vs Placebo_0 | 0.827 |  |  |  |
| -> direct |  | -0.317 | -0.981 | 0.364 |
| -> indirect |  | -0.401 | -0.92 | 0.09 |
| -> MBNMA |  | -0.376 | -0.776 | 0.018 |
|  |  |  |  |  |
| CT_250 vs Placebo_0 | 0.642 |  |  |  |
| -> direct |  | -0.096 | -0.805 | 0.612 |
| -> indirect |  | -0.176 | -0.505 | 0.151 |
| -> MBNMA |  | -0.167 | -0.456 | 0.122 |
|  |  |  |  |  |
| AE_250 vs Placebo_0 | 0.172 |  |  |  |
| -> direct |  | -0.962 | -1.855 | -0.031 |
| -> indirect |  | -0.214 | -0.453 | 0.042 |
| -> MBNMA |  | -0.268 | -0.519 | -0.022 |

**Supplementary 3.1 Forest plot**

**
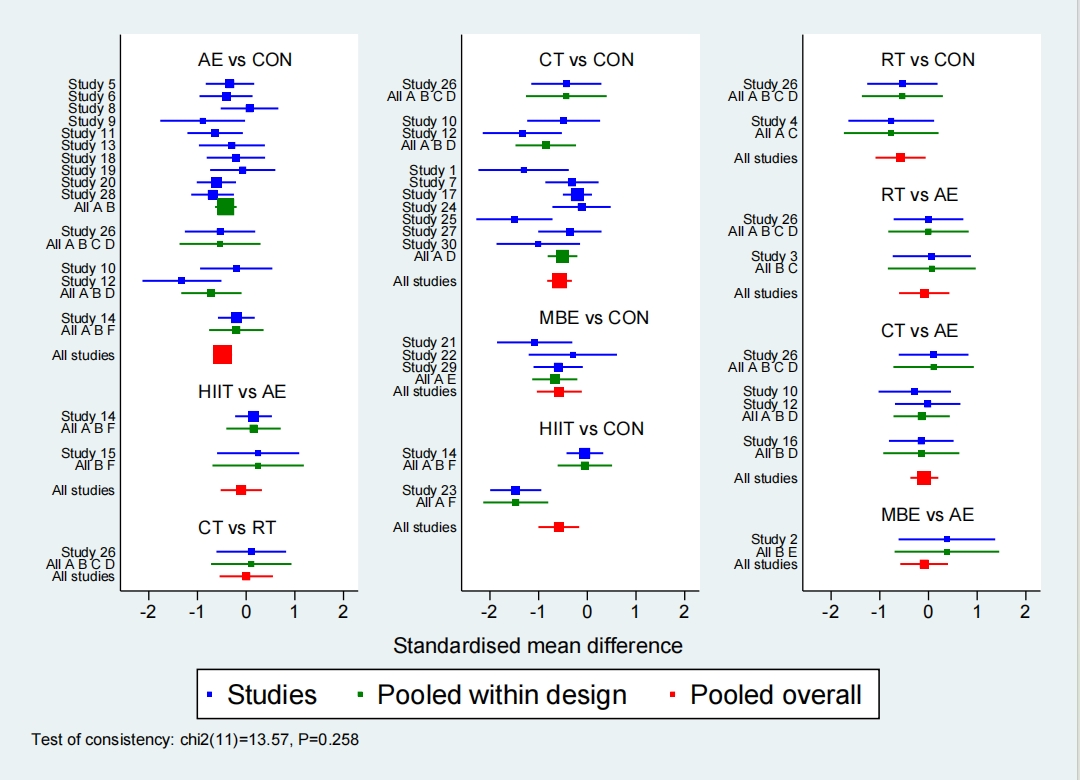
**

- **Supplementary 3.2 Details for Node-splitting**

| **Comparison** | **Direct Effect** | **Direct (SE)** | **Indirect Effect** | **Indirect (SE)** | **Network Effect (Difference)** | **Difference (SE)** | **P-value** | **tau** |
| --- | --- | --- | --- | --- | --- | --- | --- | --- |
| **CON vs AE** | -0.43 | 0.11 | -0.85 | 0.27 | 0.42 | 0.29 | 0.14 | 0.26 |
| **CON vs RT** | -0.64 | 0.35 | -0.49 | 0.41 | -0.15 | 0.54 | 0.78 | 0.28 |
| **CON vs CT** | -0.58 | 0.14 | -0.51 | 0.35 | -0.07 | 0.38 | 0.85 | 0.28 |
| **CON vs MBE** | -0.67 | 0.26 | -0.09 | 0.58 | -0.58 | 0.64 | 0.37 | 0.27 |
| **CON vs HIIT** | -0.67 | 0.26 | -0.38 | 0.42 | -0.29 | 0.49 | 0.55 | 0.28 |
| **AE vs RT** | 0.03 | 0.34 | -0.29 | 0.43 | 0.32 | 0.55 | 0.56 | 0.27 |
| **AE vs CT** | -0.08 | 0.23 | -0.09 | 0.20 | 0.01 | 0.30 | 0.98 | 0.28 |
| **AE vs MBE** | 0.38 | 0.57 | -0.20 | 0.28 | 0.58 | 0.64 | 0.37 | 0.27 |
| **AE vs HIIT** | 0.18 | 0.27 | -0.53 | 0.34 | 0.71 | 0.43 | 0.10 | 0.26 |
| **RT vs CT** | 0.11 | 0.46 | -0.06 | 0.36 | 0.17 | 0.58 | 0.77 | NA |

- **Supplementary 3.3 Funnel plot**

**
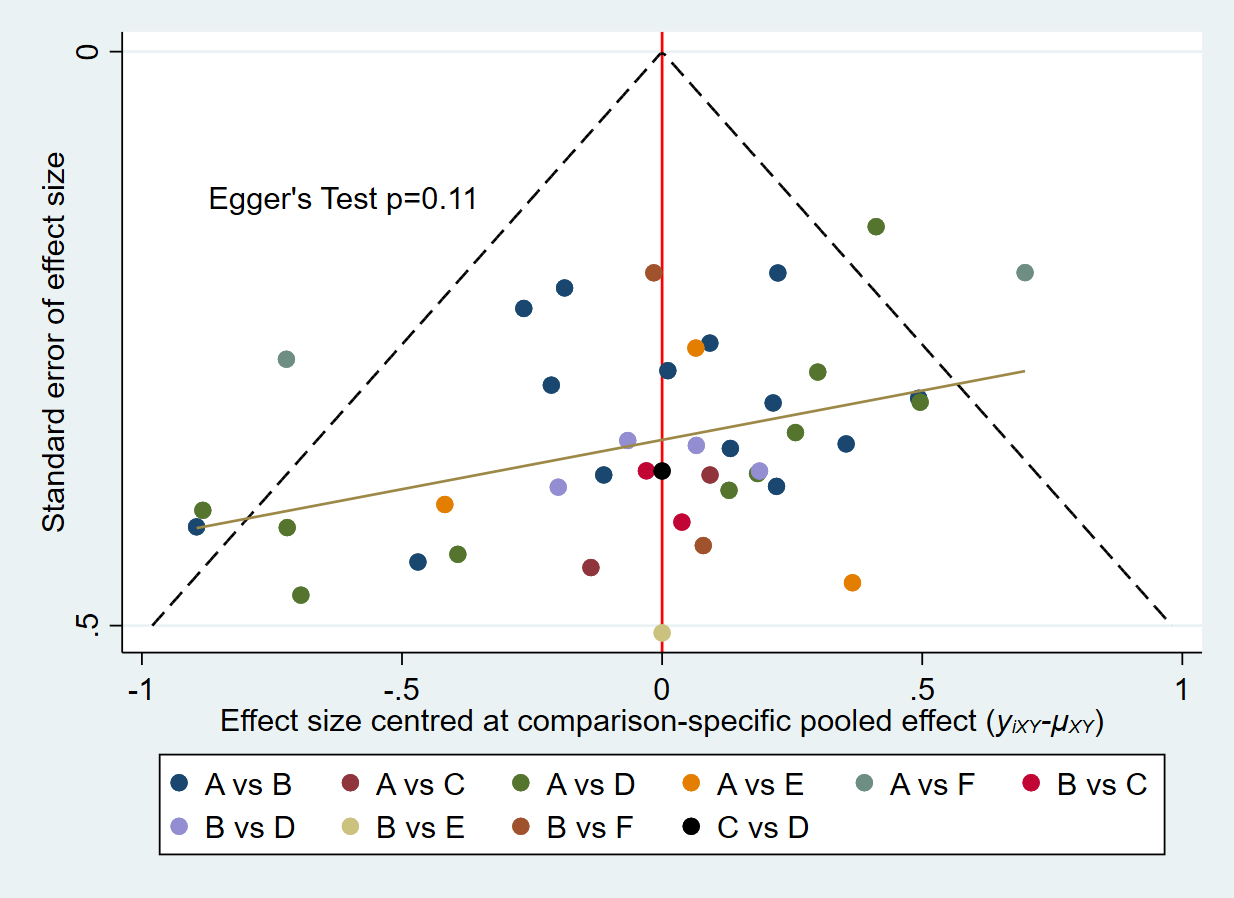
**

- **Supplementary 3.4 Loop-specific plot**


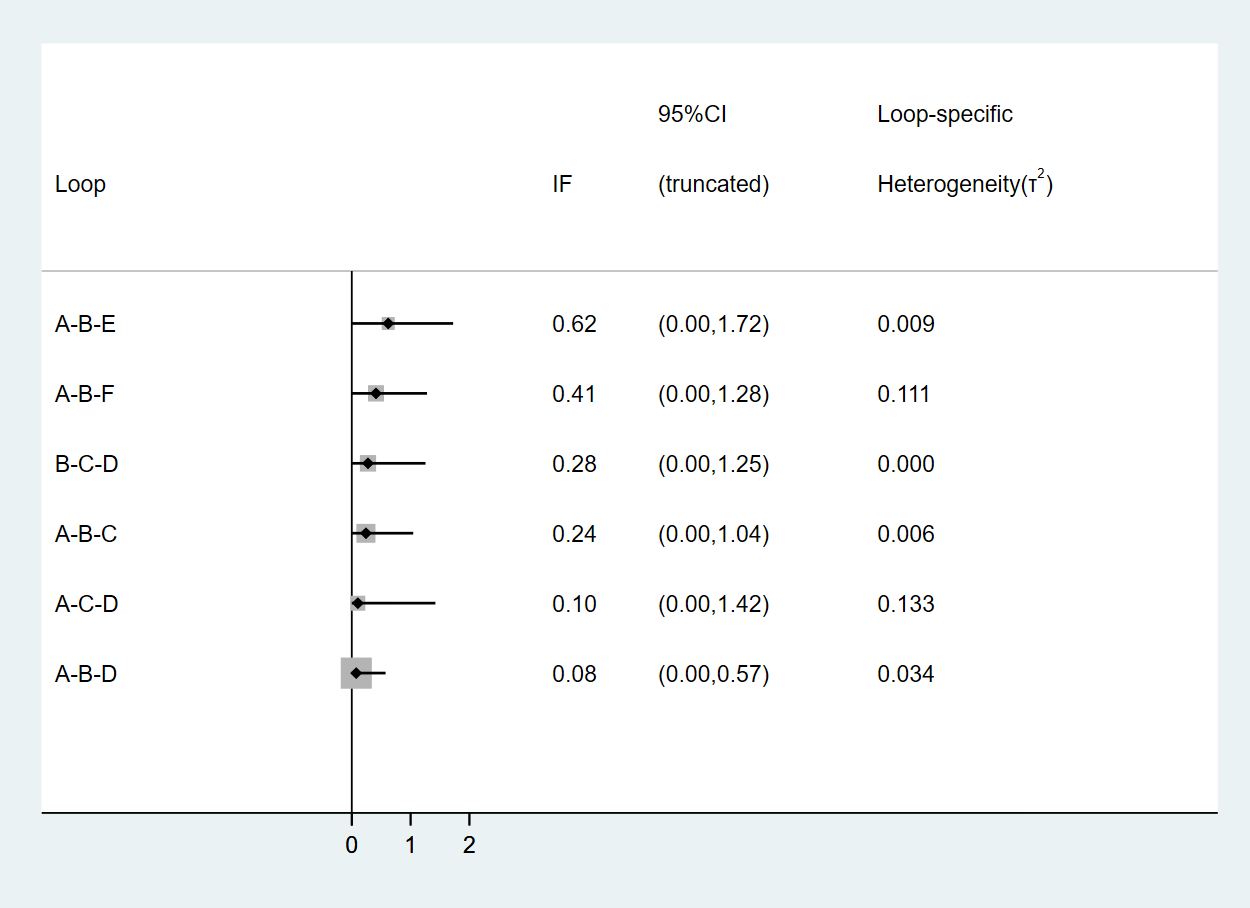


- **Supplementary 3.5 Meta regression results**

| Covariate | Shared beta  (Median and 95% CrI) | Heterogeneity standard deviation (median and  95% CrI) | % of  variance  explained |
| --- | --- | --- | --- |
| None | - | 0.30(0.16;0.48) | - |
| Mean age | 0.23(-0.1;0.60) | 0.29(0.15;0.46) | 7% |
| LVEF | 0.15(-0.20;0.53) | 0.31(0.17;0.52) | -7% |

- **3.5.1 Mean age**

When the model was adjusted for centering value of mean age 62.08, compared with the control group, the SMD value of physical activity types did not change significantly. The hierarchy from the unadjusted model retained.


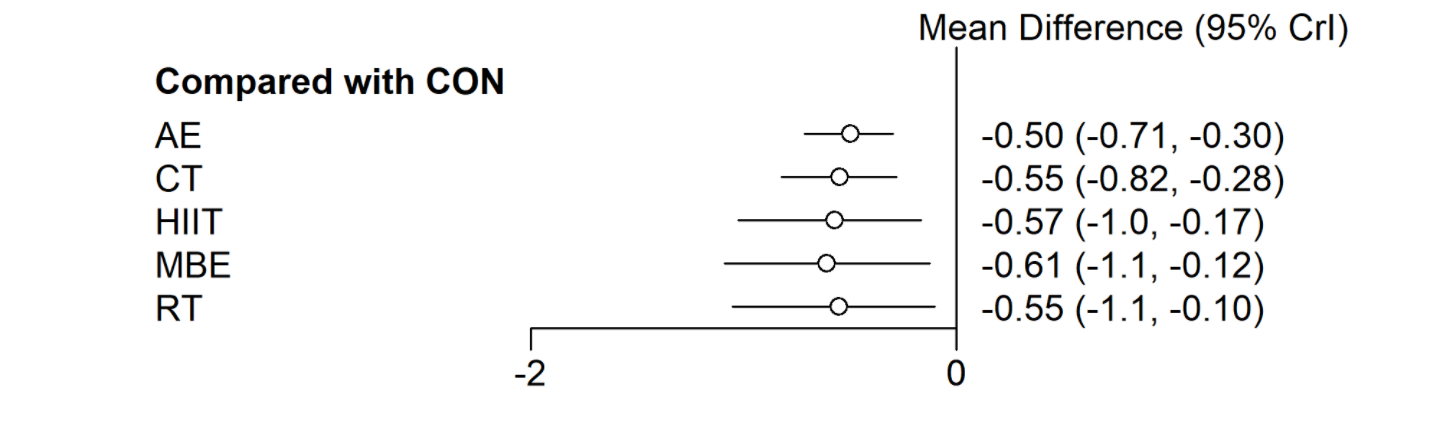


- **3.5.2 LVEF**

When the model was adjusted for centering value of LVEF 37.14, compared with the control group, the SMD value of physical activity types did not change significantly. The hierarchy from the unadjusted model retained.


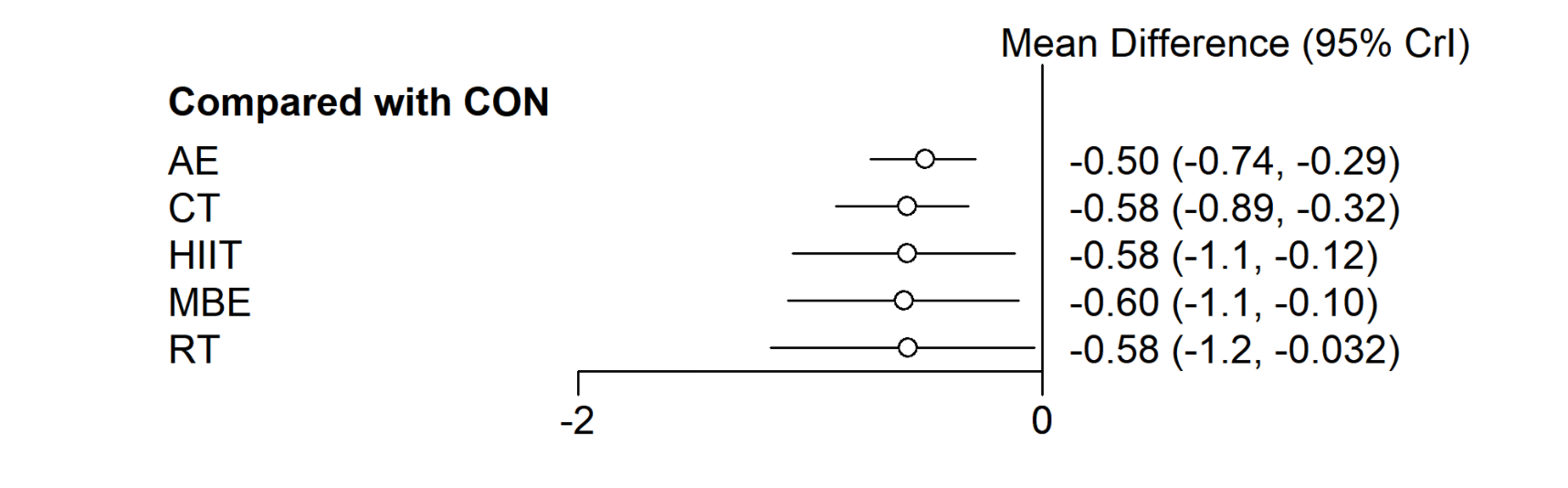

Supplement: Supplementary file 1 [file Datasheet1.doc]
